# Shared decision-making in healthcare in mainland China: a scoping review

**DOI:** 10.3389/fpubh.2023.1162993

**Published:** 2023-09-07

**Authors:** Xuejing Li, Dan Yang, Meiqi Meng, Junqiang Zhao, Yiyi Yin, Hefang Wang, Xiaoyan Zhang, Qian Liu, Mengdi Li, Jianping Liu, Yufang Hao

**Affiliations:** ^1^School of Nursing, Beijing University of Chinese Medicine, Beijing, China; ^2^Beijing University of Chinese Medicine Collaborating Center of Joanna Briggs Institute, Beijing, China; ^3^Beijing University of Chinese Medicine Best Practice Spotlight Organization, Beijing, China; ^4^School of Nursing, University of Ottawa, Ottawa, ON, Canada; ^5^Center for Research on Health and Nursing, University of Ottawa, Ottawa, ON, Canada; ^6^Center for Evidence-Based Chinese Medicine, Beijing University of Chinese Medicine, Beijing, China

**Keywords:** shared decision-making, Ottawa decision support framework, healthcare, patients decision aids, decisional needs

## Abstract

**Background:**

Shared decision-making (SDM) facilitates the participation of healthcare professionals and patients in treatment decisions. We conducted a scoping review to assess SDM’s current status in mainland China, referencing the Ottawa Decision Support Framework (ODSF).

**Methods:**

Our review encompassed extensive searches across six English and four Chinese databases, and various gray literature until April 30, 2021. Results were synthesized using thematic analysis.

**Results:**

Out of the 60 included studies, we identified three key themes based on the ODSF framework: decisional needs, decision support, and decisional outcomes. However, there appears to be a lack of comprehensive understanding of concepts related to decisional needs in China. Only a few studies have delved into feasibility, preference, choice, and outcome factors in the SDM process. Another challenge emerges from an absence of uniform standards for developing patient decision aids (PDAs). Furthermore, regarding health outcome indicators, their predominant focus remains on physiological needs.

**Conclusion:**

SDM is in its infancy in mainland China. It is important to explore the concept and expression of decisional needs in the context of Chinese culture. Subsequent studies should focus on constructing a scientifically rigorous and systematic approach for the development of PDAs, and considering the adaptation of SDM steps to the clinical context in China during SDM implementation. Concurrently, The focus on health outcomes in Chinese SDM studies, driven by the unique healthcare resource landscape, underscores the necessity of prioritizing basic needs within limited resources.

**Systematic review registration:**

https://inplasy.com/?s=202130021.

## 1. Introduction

Shared decision-making (SDM) has been increasingly advocated as an ideal approach to clinical practice (1). Patient engagement in the decision-making process has numerous benefits, including decreased decisional conflict, increased patient knowledge, and improved health outcomes such as patient satisfaction and quality of life (2, 3). The introduction of SDM in China can be traced back to 1998 (4). Since the advent of the 21st century, the long-established physician-led paternalistic medical decision-making model in China has gradually transformed into a patient-centered shared decision-making model involving both patients and physicians (5, 6). Preliminary research indicates that in recent years, SDM has been gradually gaining attention and experiencing a certain level of application in mainland China, covering areas such as diagnosis, treatment, screening, and care models (7–13). In contrast, across the international landscape, SDM has gained recognition as a prominent trend in Western societies, serving as the prevailing model for facilitating patient-centered healthcare decisions (14) and achieving patient-value based care (15). SDM studies conducted in Western countries have generated a wealth of valuable resources, including not only theoretical models (16) but also practical tools (17). Some models (1, 18) and tools (19–21) have been empirically tested and possess strong guidance and applicability. However, the significant differences in healthcare systems and cultures between Western countries and China raise questions about the feasibility of simply transferring Western SDM products into China (22). For instance, in mainland China, the uneven distribution of medical resources and disparities between patient expectations and reality poses challenges to patient participation in medical decision-making (23). These challenges further exacerbate distrust and dissatisfaction among some patients with the diagnosis and treatment process (24, 25). Limited medical knowledge, varying educational levels among patients, and regional economic disparities further complicate the implementation of SDM (26). To accurately identify the current hotspots and gaps in SDM research, a comprehensive summary of SDM-related research in China at this stage is necessary. A systematic review conducted by Rongchong Huang et al. evaluated the relevant research on SDM in China, including trials and surveys (27). While the findings of this study were disappointing, they provided a groundbreaking overview of the current status and barriers of SDM research conducted in China at that time. To date, there has been no published review specifically focusing on SDM-related research in mainland China.

To understand the current state of research in the field of SDM in mainland China, we reviewed whether research in mainland China was included in systematic reviews covering SDM concepts. When studying a complex and new field, choosing an appropriate theoretical framework is essential. The framework can facilitate conceptualizing the topics covered by the field in question and comprehensively guide the research direction (28). The Ottawa Decision Support Framework (ODSF) conceptualizes the support patients, families, and their practitioners need when deliberating about options (29–31). It guides practitioners and researchers in assessing participants’ decisional needs, providing decision support interventions, and evaluating the effects of decision support on decisional outcomes. In the context of decision-making, the focus of a needs assessment is on identifying: what a patient population needs to make better decisions; and what a population of health practitioners needs to improve the support they provide to patients during decision-making. In 2020, a systematic review (31) of decision needs included 45 studies covering research from seven countries, but we have yet to find evidence from China. A Cochrane systematic review in 2018 determined the effectiveness of interventions for increasing the use of SDM by healthcare professionals (2). It included 87 studies. The four most represented countries were the United States, Germany, Canada, and the Netherlands. None of the studies were from mainland China. Studies related to SDM are typically designed to investigate how SDM might improve outcomes that are relevant to patients. Many measurement approaches to SDM have been developed (32–34). The integration and summary of outcome indicators have been suggested to standardize subsequent studies. Shay and Lafata included 41 relevant studies in their integration of outcome information from studies related to SDM and developed a framework of SDM outcome indicators (35). Gärtner FR used this framework in his 2018 review and included 51 studies (36). However, no Chinese studies were identified in either of these systematic reviews.

It is evident that there is still a lack of studies that systematically present the current status of SDM research in China, and no eligible Chinese studies have been included in any of the systematic reviews published in recent years. Comparing the current status of SDM implementation in China with the international status may help us find implementation strategies and address these gaps. Given that the three core structures of ODSF cover a significant portion of the current focus of SDM-related research and it is one of the most widely applied SDM theoretical frameworks internationally (30), this study provides a comprehensive and systematic mapping of SDM research in mainland China from the dimensions of decisional needs, decision support, and decisional outcomes, guided by this framework. Additionally, due to the broad nature of our research question, the SDM field being in its early stages in mainland China with limited research available, and our anticipated research design and methods (37), we conducted a scoping review instead of a systematic review.

## 2. Methods

Scoping studies are an increasingly popular approach to reviewing health research evidence. To guide authors undertaking scoping studies, Arksey and O’Malley (38) developed a five-stage methodological framework: (1) identifying the research question, (2) searching for relevant studies, (3) selecting studies, (4) charting the data, (5) collating, summarizing, and reporting the results. This framework provided an excellent methodological foundation. The study findings will be reported following the Preferred Reporting Items for Systematic reviews and Meta-Analysis extension for Scoping Reviews (PRISMA-ScR) reporting guidelines (39). The protocol was registered prospectively in the Inplasy (202130021). The Supplementary material provides the PRISMA-ScR checklist (Supplementary file S1).

### 2.1. Stage 1: identifying the research question

The purpose of this study is to describe the current research on SDM in mainland China. Based on the ODSF, the objective of this scoping review is to map accessible research literature to answer the research questions systematically: (1) What were the general characteristics of SDM studies in mainland China? (2) What decisional needs of patients were focused on in these studies? (3) What SDM decision support (SDM theories, process, tools) were described to address the healthcare decisional needs? and (4) What SDM outcomes were reported?

### 2.2. Stage 2: identifying relevant studies

With the assistance of an experienced research librarian, we searched six English databases: PubMed, the Cumulative Index to Nursing and Allied Health Literature (CINAHL), EMBASE, Cochrane Library, PsycINFO and Web of Science. We also searched four Chinese databases: China National Knowledge Infrastructure, WanFang Database, VIP Database, and China Biology Medicine. All databases were searched from 1968 (this year was chosen as the starting year due to the prevalence of SDM publications since that time (40)) to April 2021. The retrieval also included unpublished gray literature. Gray literature were searched using Baidu Scholar^1^ and Google scholar. These strategies will ensure that documents on SDM in mainland China are identified in search of the literature. The finalized search strategy is shown in Appendix 1.

### 2.3. Stage 3: study selection

This study will integrate all the studies on SDM in mainland China as thoroughly as possible, so we developed relatively broad initial criteria inclusion criteria based on clearly identifiable populations, concepts, and contexts (PCC) (41). Table 1 illustrates how the PCC acronym was applied. In addition to the PCC, we also limited the study design and language in the inclusion criteria.

**Table 1:** Inclusion criteria.

Population	Studies involving Chinese patients or population who has participated in SDM-related research with no restrictions on age or gender.
Concept	On the premise of the concept of joint participation of healthcare providers and patients in designated healthcare decisions, we included all studies on the measurement of decisional needs, the design and application of processes and tools related to decision support, and the evaluation of the effectiveness of SDM implementation.
Context	We focus only on studies conducted in mainland China, with no restrictions on the nationality or location of the research team.
Study design	Cross-sectional, qualitative, mixed-method, cohort, case–control, case study, and RCT. (Excluded research types: review, opinion, news, comment, and research that can only obtain abstract)
Language	There were no restrictions on the language included in the literature in this study.

Study selection was performed in several steps. First, after de-duplication, review authors (DY and XJL) double-screened all titles and abstracts in duplicate using NoteExpress 3.2.0 document management software. Then, reviewers (DY and XJL) double-screened the full text for all studies included. Disagreements were further discussed by the third reviewing author (YFH) to reach a consensus.

### 2.4. Stage 4: data collection

The literature screening gave us a general overview of the objectives of the studies. We found two main kinds of SDM studies conducted in China: descriptive studies focusing on decisional needs and influencing factors of SDM; and analytical studies focusing on the construction of SDM-related tools and the effects of SDM implementation. Because of the significant differences in the presentation of information between these two kinds of studies, we developed two content extraction forms using Excel. In the descriptive study, we extracted: study identifications, concepts, methods, theories, models utilization, and outcomes. For the analytical study, we mainly extracted: study identifications, concepts, methods, patient characteristics of the intervention group, patient characteristics of the control group, theories and models utilization, decision support tools, SDM process, outcomes, and influencing factors.

### 2.5. Stage 5: collation, summary and reporting of the results

This stage involves collating, summarizing and reporting the results of the selected evidence, which was undertaken by the whole research team in an iterative process until consensus on the thematic results and implications of the scoping review were agreed. We used two main strategies to analyze the data in this study: (1) descriptive number statistics for general information about the included literature and (2) qualitative thematic analysis.

In the first step, we employed a framework analysis approach to map the extracted data into the three core concepts of the Ottawa Decision Support Framework (ODSF), namely Decisional needs, Decisional support, and Decisional Outcomes. Subsequently, a comprehensive analysis of the three core concepts was conducted. Step 2: Analyze the content of Decisional needs (31). The ODSF Decisional needs Coding Manual (31) was used as the primary coding tool to conduct a thematic analysis for the classification of decisional needs. Step 3: The content of Decision support was divided into two parts, the SDM process and decisional support tools. In the SDM process, we classified the implementation process of SDM based on the Interprofessional Shared Decision Making (IP-SDM) Model (42), and classified the doctor-patient conversation time in the SDM process using the three-talk model (43). In the decisional support tools section, we organized the extracted Patient Decision Aids (PDAs) construction process according to the National Health Service Standards framework for shared decision-making support tools (NHS Standards framework) (44). Step 4: Outcomes were classified into three categories: cognitive-affective, behavioral, and health outcomes, according to Shay and Lafata’s classification model (35) for outcomes in SDM-related studies.

The lead author (XL) wrote the scoping analysis, which was then checked independently by three collaborators (DY, MM, and JZ) who fed back their comments. Any disagreements were discussed and resolved.

## 3. Results

### 3.1. Basic characteristics

**Figure 1:**
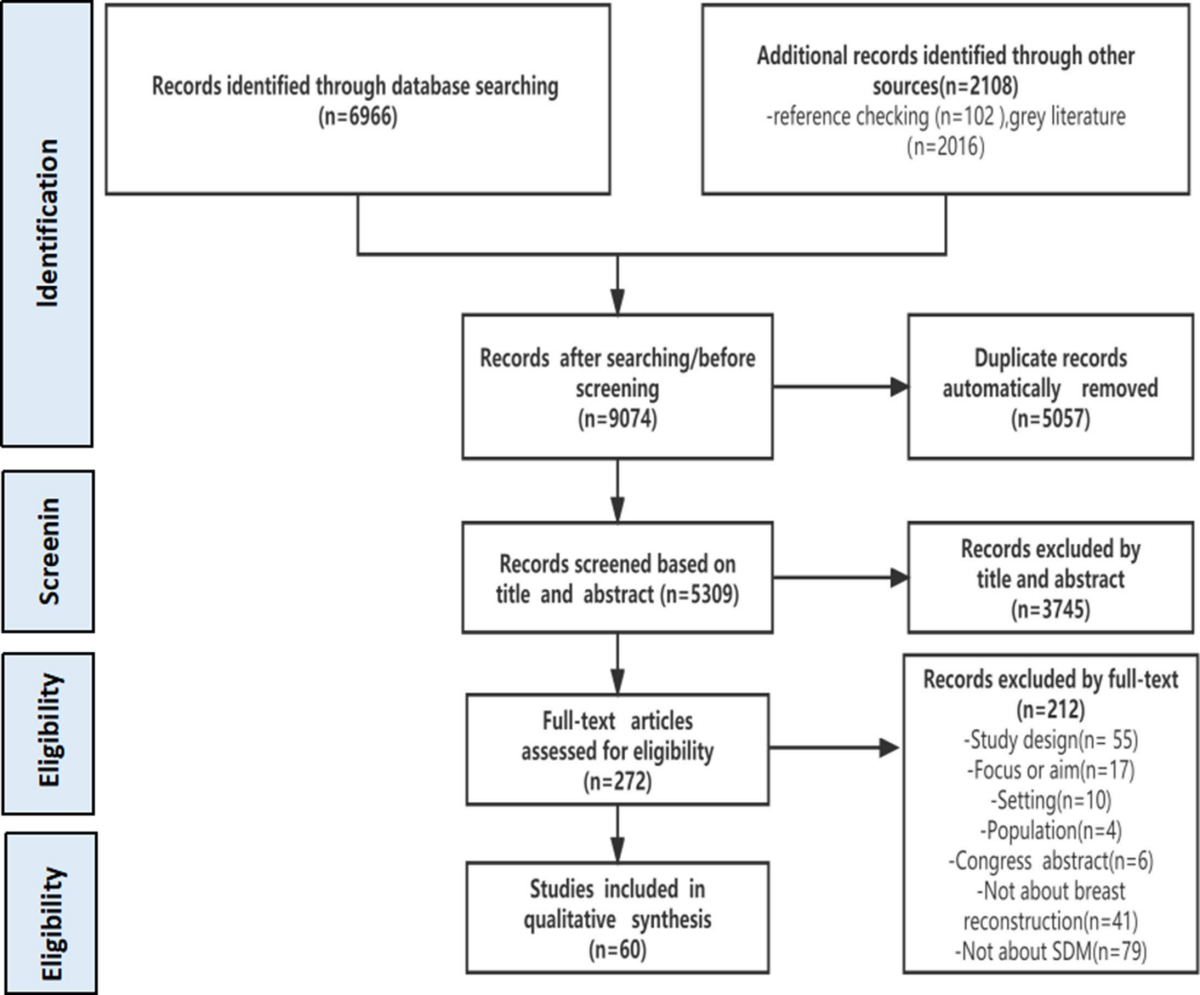
Scoping review flowchart.

Of the 6,966 and 2,108 records retrieved from the database and gray literature search, 60 papers were finally included after screening (see Appendix 2 for a list of included studies). The literature search and screening diagram are shown in Figure 1. The basic characteristics of those 60 papers are presented in Table 2. According to the research contents, the 60 papers included were divided into two main categories: one is descriptive research focusing on decision needs and influencing factors of patient participation in decision-making (29/60, 48.33%); the other is decision support focusing on the development and implementation effects of decision aids (31/60, 51.67%). For better analysis, the included studies were analyzed as a whole or in groups, according to the research questions for the review detailed above.

**Table 2:** Basic characteristics of included studies (*n* = 60).

Basic characteristics	No. of studies *n* (%)	Basic characteristics	No. of studies *n* (%)
Year of publication		Province	
2010	1 (1.67%)	Guangdong	8 (13.33%)
2013	1 (1.67%)	Hunan	8 (13.33%)
2014	2 (3.33%)	Shanghai	8 (13.33%)
2015	1 (1.67%)	Beijing	7 (11.67%)
2016	2 (3.33%)	Sichuan	4 (6.67%)
2017	5 (8.33%)	Henan	3 (5.00%)
2018	3 (5.00%)	Hubei	3 (5.00%)
2019	15 (25.00%)	Zhejiang	3 (5.00%)
2020	18 (30.00%)	Chongqing	3 (5.00%)
2021	12 (20.00%)	Jiangsu	2 (3.33%)
Language		Liaoning	2 (3.33%)
Chinese	55 (91.67%)	Shaanxi	2 (3.33%)
English	5 (8.33%)	Tianjin	2 (3.33%)
Funding support		Fujian	1 (1.67%)
National	10 (16.67%)	Hebei	1 (1.67%)
Municipal	6 (10.00%)	Heilongjiang	1 (1.67%)
Provincial	8 (13.33%)	Shanxi	1 (1.67%)
Organizational	12 (20.00%)	Anhui	1 (1.67%)
No	24 (40.00%)	Research design	
Ethics		Cross-sectional study	17 (28.33%)
No	34 (56.67%)	Randomized controlled trial	14 (23.33%)
Yes	26 (43.33%)	Non-Randomized controlled trial	13 (21.67%)
		Qualitative study	9 (15.00%)
		Mixed-method study	7 (11.67%)

Overall, the publication has shown an increasing trend in recent years, with the first publication dating back to 2010 and a greater proportion of research literature written in the previous 3 years (2019–2021). The studies were published predominately in Chinese journals (55/60, 91.67%). 36 (60%) of the included studies got funding support. Most of the included studies (56.67%) did not explicitly describe the ethical considerations. The commonly used research designs for SDM were cross-sectional study (17/60, 28.33%), randomized controlled trial (14/60, 23.33%), and non-Randomized controlled trial (13/60, 21.67%). The Types of patients included in those SDM research mainly focused on oncology (e.g., breast cancer; 18/60, 30.00%), cardiovascular diseases (e.g., atrial fibrillation; 7/60, 11.67%), chronic diseases (e.g., diabetes mellitus; 7/60, 11.67%), and mental diseases (e.g., schizophrenia; 7/60, 11.67%; see Table 3).

**Table 3:** Types of patients included (*n* = 60).

Type of patients included	No. of studies (n (%))	Type of patients included	No. of studies (n (%))
Oncology	18 (30.00%)	Mental diseases	7 (11.67%)
Breast cancer (45–50)	6 (10.00%)	Schizophrenia (51–55)	4 (6.67%)
Lung cancer (56, 57)	2 (3.33%)	Affective disorder (58–60)	3 (5.00%)
Liver cancer (7, 61)	2 (3.33%)	Orthopedic diseases	5 (8.33%)
Gynecological oncology (62)	1 (1.67%)	Total knee arthroplasty (63–66)	4 (6.67%)
Esophageal cancer (67)	1 (1.67%)	Fracture of lower limb (68)	1 (1.67%)
Gastric cancer (69)	1 (1.67%)	Ophthalmic Diseases	3 (5.00%)
Rectal cancer (70)	1 (1.67%)	Ophthalmectomy (71)	1 (1.67%)
Thyroid cancer (72)	1 (1.67%)	Age-related macular degeneration (73)	1 (1.67%)
Cancer (type not specified) (74–76)	3 (5.00%)	Exudative age-related macular degeneration (77)	1 (1.67%)
Cardiovascular diseases	7 (11.67%)	Other diseases	4 (6.67%)
Atrial fibrillation (7, 78–80)	4 (6.67%)	Neurological diseases (81, 82)	2 (3.33%)
Coronary heart disease (83–85)	3 (5.00%)	Inflammatory bowel diseases (86)	1 (1.67%)
Chronic diseases	7 (11.67%)	Dermatologic diseases (87)	1 (1.67%)
Diabetes mellitus (76, 88–90)	4 (6.67%)	Disease type not specified	9 (15.00%)
Hypertension (91)	1 (1.67%)	Outpatients and inpatients (92–96)	5 (8.33%)
Chronic renal failure (97)	1 (1.67%)	Surgery (98, 99)	2 (3.33%)
Coexistence of chronic diseases (51)	1 (1.67%)	Parturient (100)	1 (1.67%)
		ICU (95)	1 (1.67%)

### 3.2. Theory used

A total of 13 theories/models/frameworks to guide SDM studies were reported in 13 studies (21.67%) (7, 9, 45, 46, 61–63, 72, 74, 78, 79, 83, 92), among which ODSF was the most frequently used one (8/13, 61.54%) (7, 9, 46, 61–63, 72, 79) and was mainly used to guide the construction of decision aids, the implementation of decision making needs assessment and the design of the interview outline. The remaining 12 frameworks (45, 61, 72, 74, 83, 92, 93) all appeared only once in the included studies. The specific theories/models/frameworks and their role in the study are shown in Appendix 3.

### 3.3. Decisional needs

The ODSF Decisional Needs Coding Manual defines all aspects of decision requirements in detail and provides rich coding examples. Decisional needs were the focus of 56 out of the 60 included studies (93.33%). Based on the ODSF Decisional Needs Coding Manual (31), seven main themes were identified: (1) Decisional conflict; (2) Inadequate knowledge; (3) Unrealistic expectations; (4) Unclear values; (5) Inadequate support or resources; (6) Complex decision characteristics; and (7) Personal and clinical needs. Of the 56 studies, all ODSF decisional needs were reported in 1 (2%) to 33 (58.93%) studies (see Table 4). In this study, the three most frequent manifestations of decisional needs were feeling uninformed about the health problem, condition or situation to make a decision (33/56, 58.93%) (46–53, 58, 59, 61–65, 68, 70, 72, 74–77, 79, 81, 82, 87, 88, 93, 94, 98–101); lacking instrumental help (24/56, 42.86%) (9, 49, 50, 61, 62, 64–69, 71, 77, 79, 81–85, 87–89, 91, 102); feeling lacking confidence in the ability to implement chosen option (22/56, 39.29%) (46, 48–50, 53, 54, 56, 58, 61, 63, 65, 72, 75, 77, 81, 82, 84, 86, 87, 97, 99, 100) (see Appendix 4).

**Table 4:** Frequency (%) of studies reporting ODSF decisional needs.

Rank	Decisional need (code number)	*N* (%) of studies (*N* = 56)
1	Inadequate knowledge (2)	37 (66.07%)
2	Inadequate instrumental help (5.11)	24 (42.86%)
3	Inadequate self-efficacy (5.5)	23 (41.07%)
4	Inadequate emotional support (5.10)	20 (35.71%)
5	Decisional conflict (1)	16 (28.57%)
6	Inadequate information (5.8)	12 (21.43%)
7	Unclear about the role in decision making(5.3)	11 (19.64%)
8	Clinical needs (7.2)	10 (17.86%)
9	Inadequate financial assistance (5.13)	9 (16.07%)
10	Personal needs (7.1)	9 (16.07%)
11	Inadequate health/social service (5.12)	8 (14.29%)
12	Inadequate advice (5.9)	7 (12.50%)
13	Difficult decision timing (6.2)	6 (10.75%)
14	Social Pressure (5.2)	4 (7.14%)
15	Inadequate experience (5.4)	4 (7.14%)
16	Unrealistic expectations (3)	3 (5.36%)
17	Unclear values (4)	3 (5.36%)
18	lacks the ability or skill to make a decision (5.7)	3 (5.36%)
19	Difficult decision type (6.1)	3 (5.36%)
20	Inadequate motivation (5.6)	2 (3.57%)

#### 3.3.1. Decisional conflict

A variety of experiences triggered a series of decisional conflicts faced by patients during the SDM process (16/56,28.57%). Uncertainty of action emerged when their choices were challenged by risk, loss, regret, and personal life values. Five studies (48, 57, 61, 84, 96) (5/56,8.93%) reported that, when involved in decision-making, patients often described that they faced decision difficulties because they were unsure of what is the best course of action. In addition, other common manifestations of patient decision-makers in decisional conflict are worrying about possible problems (7/56,12.50%) (45, 46, 48, 54, 73, 80, 93) or feeling upset in making decisions (7/56,12.50%) (45, 62–65, 80, 98).

#### 3.3.2. Inadequate knowledge

Patient decision-makers in 37 studies (37/56, 66.07%) identified inadequate knowledge in their decision-making. In these studies, the most reported manifestation (49, 56, 70, 76, 77, 84, 86, 98, 99) of this need was feeling uninformed about the health problem, condition, or situation to attempt a decision (33/56, 58.93%), followed by lacking awareness of what decision needs to be made (9/56, 16.07%) and feeling uninformed enough about options (56, 73, 86) (3/56, 5.36%).

#### 3.3.3. Unrealistic expectations and unclear values

Few studies (64, 77, 90) (3/56, 5.36%) found that patients reported unrealistic expectations in the decision-making process. These participants reported that they did not know the odds of benefits versus harms and described that it is hard to believe that the outcome probabilities applied to them. Moreover, in three studies (45, 70, 90) (3/56, 5.36%), patient decision-makers reported feeling unclear values.

#### 3.3.4. Inadequate support or resources

Most studies (51/56, 91.07%) have reported that patient decision-makers feel inadequately supported and resourced in the decision-making process. Forty-five of these studies reported a lack of social support for patient decision-makers when making or implementing decisions. The most common manifestation (9, 49, 50, 61, 62, 64–69, 71, 77, 79, 81–85, 87–89, 91, 102) (24/56, 42.86%) was lacking tools to help patient decision-makers in making and implementing decisions, although some studies mentioned that patient decision aids had been used in some patient decisions. Other manifestations are lacking access to information, advice from significant others involved in the decision, and emotional support from others. Twenty-three of these studies (45, 46, 48, 50, 53, 54, 56, 58, 61, 63, 65, 72, 75, 77, 82, 84, 86, 87, 97, 99) (41.07%) reported a lack of internal resources for patient decision-makers in making or implementing decisions. Many of them lacked self-efficacy, mainly in terms of lacking confidence in their ability to participate in decision-making and in implementing the chosen option.

#### 3.3.5. Complex decision characteristics

Complex decision characteristics are composed of three main types of decisional needs: difficult decision types, difficult decision timing, and unreceptive decision stages. Of 56 studies, only three studies (76, 78, 96) (3.57%) mentioned difficult decision types. Only six studies (45, 48, 51, 54, 73, 77) of 56 studies (6/56 10.71%) reported difficult decision timing, including delays in decision times that exacerbated surgical patients’ uncertainty about their surgical options (48, 77) and tight decision times that made it difficult for healthcare workers to communicate in detail with each patient (51, 54).

#### 3.3.6. Personal and clinical needs

Of 56 studies, 19 (19/56, 33.93%) describe at least one personal or clinical need of patient decision-makers. In terms of individual needs, one study ^[49]^highlighted the need to consider demographic characteristics, and eight (45, 47, 54, 58, 61, 75, 77, 91) studies described tailored information, such as information on medication use and self-management that matched patient preferences. Furthermore, In terms of clinical needs, 10 studies (49, 63, 64, 66, 67, 70, 78, 82, 84, 97) reported the need to tailor decision support to patients’ psychological perceptions, health status, emotional state, and preferences when providing decision support.

### 3.4. SDM process

#### 3.4.1. Shared decision-making implementation process

**Figure 2:**
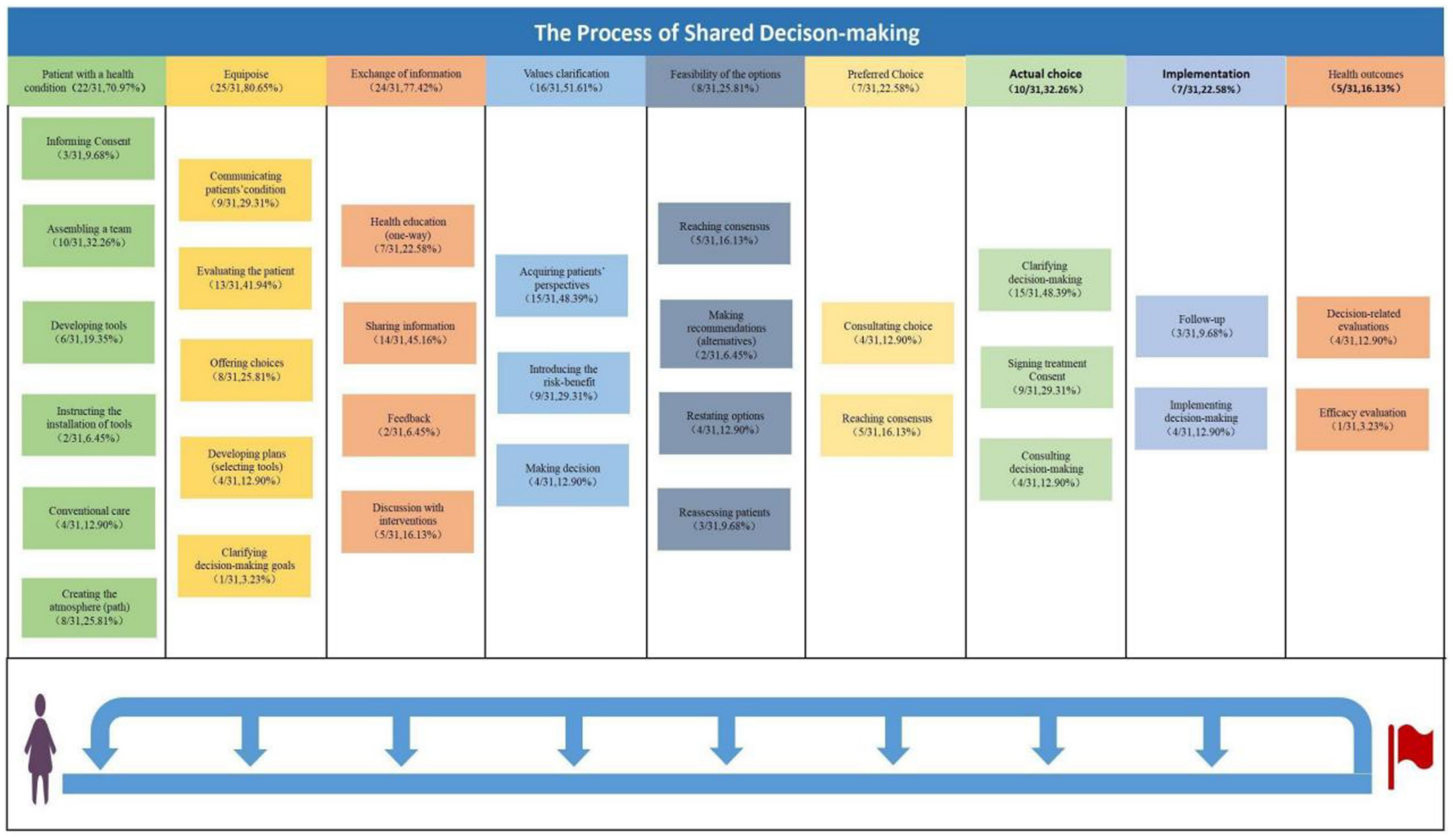
The process of SDM.

IP-SDM model takes into account the involvement of multiple professionals in the SDM process, including patients, physicians, and other healthcare workers. In this study, it helps us to understand and classify the implementation process of SDM. The implementation process of shared decision-making is exemplified by 31 papers. We mapped the themes according to the IP-SDM model. According to the core constructs of the IP-SDM model, we divided the SDM process into nine steps, of which 22 studies (22/31,70.97%) (7, 50, 52, 53, 59, 61–68, 79, 81–85, 91, 97, 102) referred to “patient with a health condition.” We subdivided this theme into informing consent (3/31,9.68%) (59, 83, 102), assembling a team (10/31,32.26%) (50, 53, 59, 64–67, 81, 84, 91, 97), developing tools (6/31,19.35%) (50, 59, 62, 85, 91, 92), instruction for installation of tools (2/31,6.45%) (7, 62) conventional care (4/31,12.90%) (52, 53, 63, 68), creation of atmosphere (8/31,25.81%) (59, 61, 67, 79, 81–84). Please refer to Appendix 5 for references related to these steps. Figure 2 shows the corresponding results.

#### 3.4.2. Participants in the SDM process

The role of the implementer was mentioned in all 31 studies on the shared decision-making process and 27 studies (27/31,87.10%) (9, 49, 50, 52, 53, 57, 59–69, 71, 79, 82–84, 87–91, 97, 102) mentioned the shared decision-making implementer’s role as physician. In 22 studies (22/31,70.97%) (9, 49, 52, 57, 59, 61–64, 66–69, 81, 83, 84, 87, 91, 97) the implementer was nurse, in nine studies (9/31,29.03%) (49, 62, 67, 69, 81–83, 91, 97) the implementer was nursing managers, four studies (4/31,12.90%) (9, 61, 79, 84) in which the implementer was the member of the research team, three studies (3/31,9.68%) (49, 84, 91) in which the implementer was mentioned as a medical manager, and two studies (2/31,6.45%) (50, 62) in which the implementer was a rehabilitator or technician. Information technology personnel (62), counselors (49), and volunteers (49) were mentioned as implementers in one study each (1/31,3.23%). In addition to the above population, patients and their families were also involved in the shared decision-making. A total of 1876 patients and their families were included in 31 studies, of which 29 (29/31,93.55%) mentioned the involvement of patients and 20 (20/31,64.52%) mentioned the involvement of patients’ families.

#### 3.4.3. Timing of conversations in the SDM process

**Figure 3:**
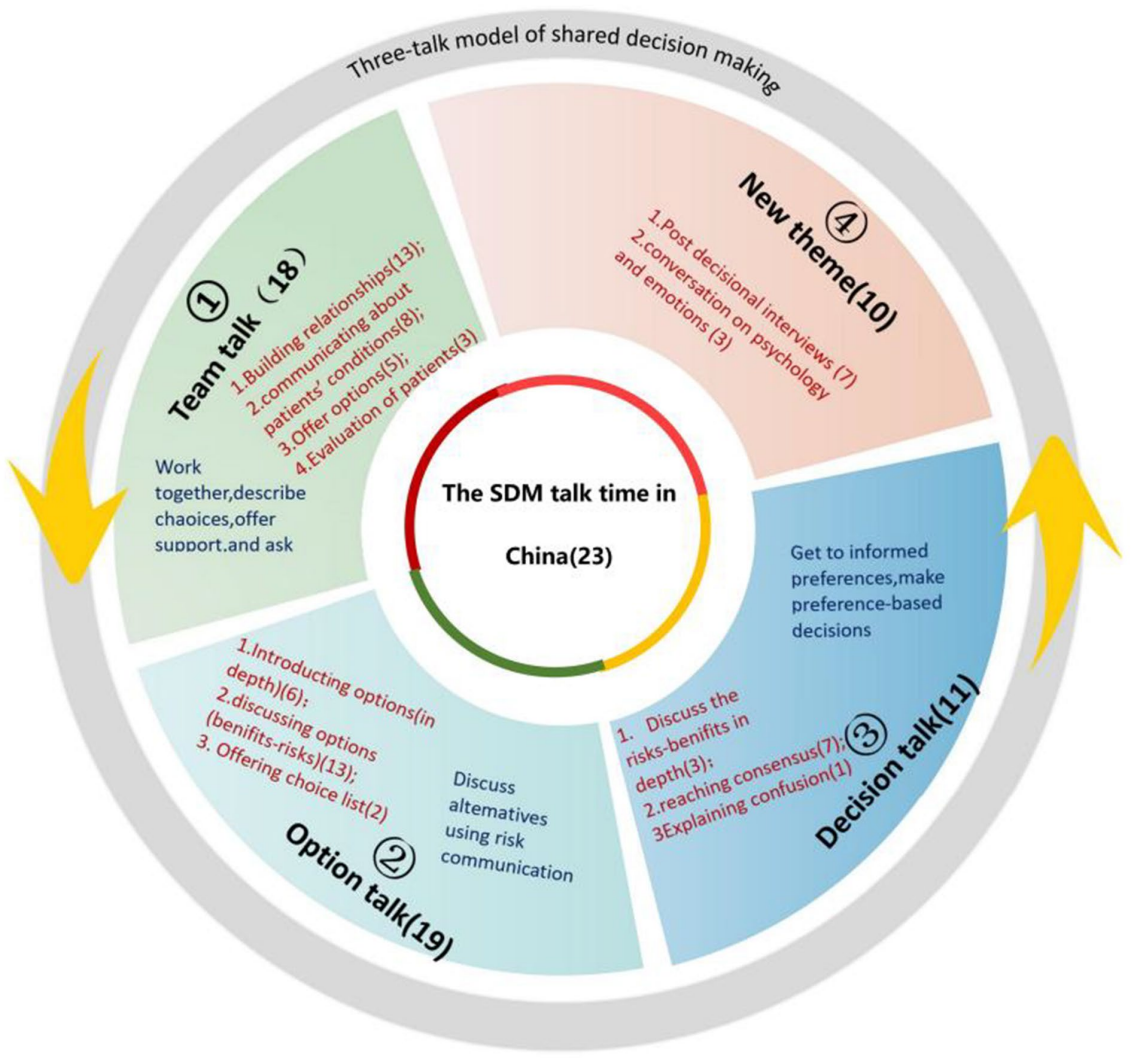
The SDM talk time in China.

The doctor-patient communication process is an essential component of SDM. The three-talk model divides doctor-patient communication into three steps: information exchange, emotion handling and decision making. Twenty-three studies referred to (23/31) conversation timing categorized according to the three core constructs of the three-talk model, of which 18 studies (18/31) (50, 52, 53, 59–62, 65–68, 71, 79, 81, 82, 84, 87–89, 91, 102) are related to team talk. Team talk-related content was divided into establishing relationships (13/31,41.94%) (50, 57, 59, 63, 65, 66, 79, 81, 84, 87, 89, 91, 102); communicating patients’ condition (8/31,25.81%) (50, 52, 53, 59–62, 65–68, 79, 81, 84, 87, 89, 91, 102); offering options (5/31,16.13%) (61, 68, 81, 84, 102); evaluating patients (3/31,9.68%) (57, 59, 63). Nineteen studies mentioned option talk (19/31) (49, 52, 53, 57, 59–61, 63–68, 79, 81, 84, 87, 91, 97) were divided into introducing options (in-depth; 6/31,19.35%) (64–67, 79, 87); discussing options (risk–benefits; 13/31,41.94%) (49, 52, 53, 57, 59, 60, 63, 66, 68, 81, 84, 91, 97); offering choice list (2/31,6.45%) (61, 84). Eleven studies mentioned decision talk (11/31) (50, 53, 59–63, 67, 84, 87, 97) were related to discussing risk–benefits (in-depth)(3/31, 9.68%) (53, 59, 87), reaching consensus (7/31, 22.58%) (60–63, 67, 84, 97), explaining confusions (1/31,3.23%) (50). Ten studies (10/31) (52, 59, 62, 64–68, 79, 89) addressed new themes that could not be categorized, including post-decisional interviews (7/31,22.58%) (62, 64–67, 79, 89), conversations on psychology and emotions (3/31, 9.68%) (52, 59, 68). See Figure 3 for a detailed description.

### 3.5. Shared decision-making support tools

#### 3.5.1. Name and form of SDM support tools

Thirty studies mentioned the presence of the interventions, including online tools, paper booklets, and courses. Seven studies mentioned online tools (7/30,23.33%), of which one was an app (1/30, 3.33%) (62), two were WeChat groups (2/30, 6.67%) (49, 50), two were public websites (2/30, 6.67%) (50, 69), one was an applet (1/30, 3.33%) (7), and one was an online decision aid (1/30,3.33%) (85). Fifteen studies (15/30,50.00%) mentioned paper-based tools, of which one was a chart (1/30,3.33%) (49), one was a process picture (no specific; 1/30,3.33%) (81), 12 were booklet (12/30,40.00%) (9, 50, 61, 64–66, 84, 85, 87–89, 91), and one was a patient selection form (1/30,3.33%) (91). The interventions in 8 eight studies were in the category of courses (8/30, 26.67%), of which six were videos (6/30, 20.00%) (49, 50, 64–66, 87), and two were lectures (2/30, 6.67%) (50, 66).

#### 3.5.2. The process of developing the SDM support tools

**Figure 4:**
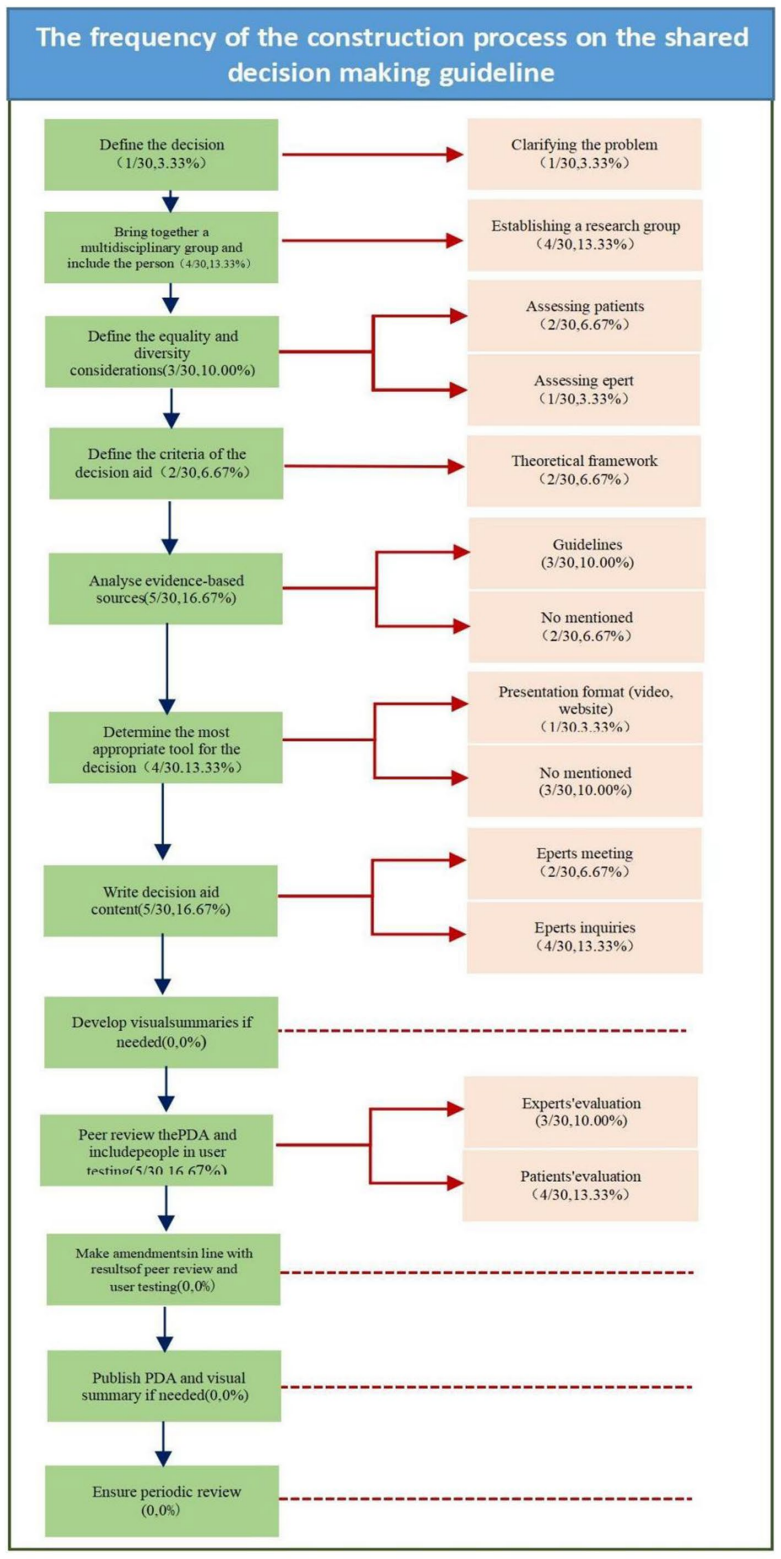
The frequency of the construction process on the SDM guideline.

We mapped the results of this part to the construction process of PDAs in NHS’s Standards framework for shared decision-making support tools, including patient decision aids. This guideline sets out a standard to support people in designing patient decision aids (PDAs). In this study, nine studies (9/30,30%) (7, 9, 49, 61, 62, 82, 84, 85, 89) were extracted from 30 articles about the interventions. There were 12 steps in the construction process in the NHS standard. However, corresponding to the standard, only 8 of these steps could be reflected in our study. For comprehensive construction procedures and relevant literature sources please refer to Appendix 5. A detailed classification is presented in Figure 4.

### 3.6. The outcomes of participation in SDM in China

**Figure 5:**
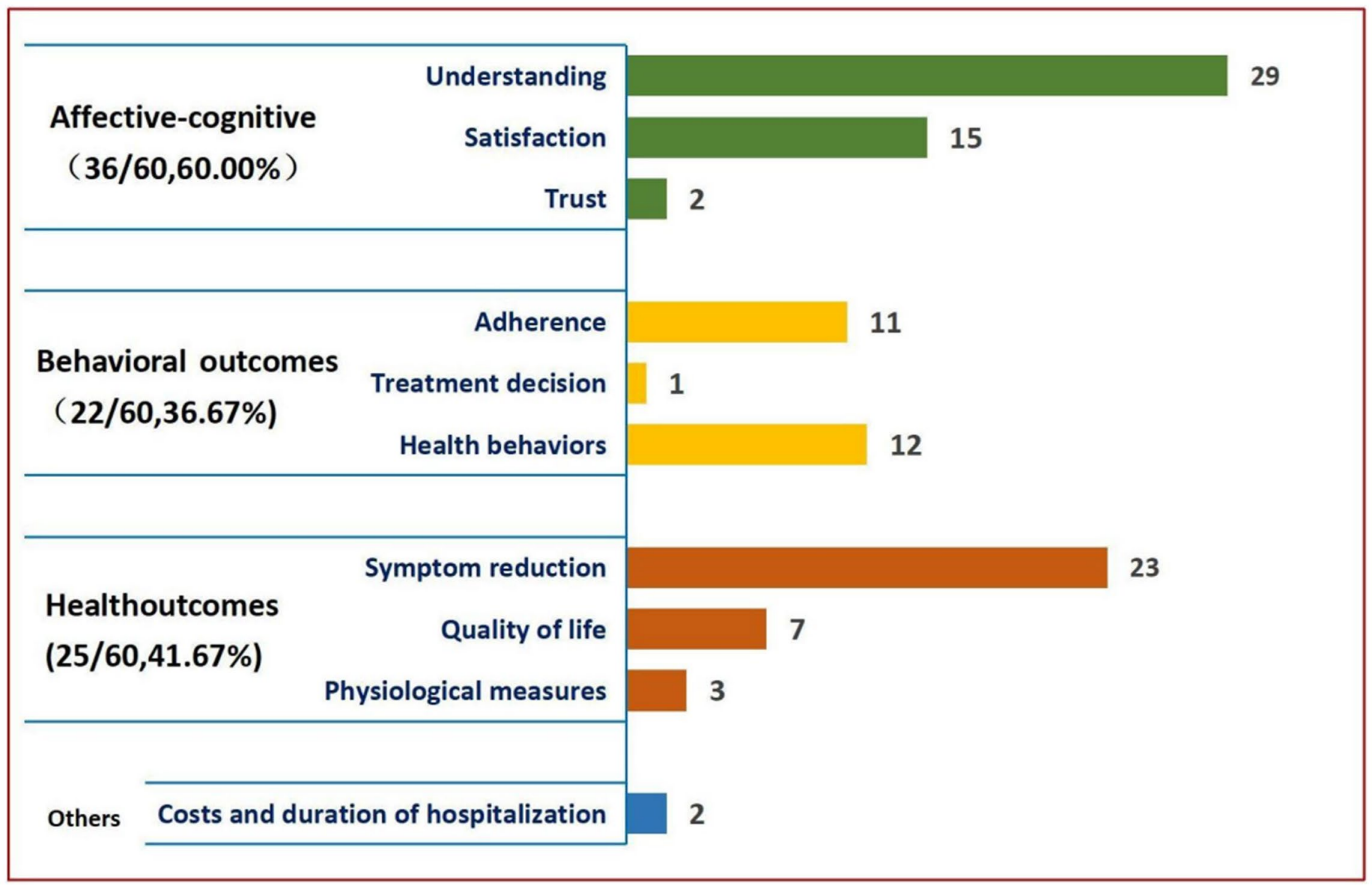
The presentation of decision related outcome analysis results.

Shay and Lafata’s classification model classifies outcomes into cognitively affective, behavioral, and health outcomes. Based on the model proposed by Shay and Lafata for the categorization analysis of decision outcome indicators, a total of 68 outcome indicators addressed in the 60 included papers were grouped into four domains of affective-cognitive outcomes (36/60,60.00%) (47, 48, 52, 55–58, 61, 62, 66–68, 70, 72, 74–78, 81, 82, 84, 86, 87, 91–102), behavioral outcomes (22/60,36.67%) (48, 49, 51, 53, 58–61, 63, 68, 69, 72, 74, 75, 81, 83, 84, 86, 87, 89, 97, 99), health outcomes (25/60,41.67%) (49, 50, 52, 53, 57, 59, 60, 62–69, 74, 78, 82, 83, 87, 88, 90, 97, 100, 102). The length of time and cost of hospitalization could not be classified in the model (2/60,3.33%) (66, 102). The specific categorization of outcome indicators is shown in Figure 5. See Appendix 6 for specific corresponding references.

## 4. Discussion

To the best of our knowledge, this is the first scoping review of SDM studies in mainland China. We systematically examined the development of SDM in healthcare in mainland China. The following discussion section is organized according to the research questions.

### 4.1. Development of SDM in mainland China

The introduction of SDM in China was influenced primarily by foreign research hotspots, and can be traced back to 1998, when ethics expert Professor Mingjie Zhao first introduced the concept of SDM to Chinese peers and emphasized the important role of patients in medical practice (6). However, the first clinical practice study of SDM in China was not published until 2015 (8). Therefore, SDM research still has much room for improvement, and the patient population needs further refinement. In addition, the absence of formally issued SDM-related policies in China’s mainland has weakened the enthusiasm of physicians to some extent. Policy support is a strong incentive for SDM implementation.

### 4.2. Few researchers in mainland China have focused on the implementation of SDM theoretical models

With the development of SDM, scholars from various countries have proposed different models to promote its implementation in medical care. As SDM research in China is still at the stage of theoretical planning and small-scale explorative applications, implementation has been limited. Only about one-fifth of the SDM studies (7, 9, 45, 46, 61–63, 72, 74, 78, 79, 83, 92) in our review used theoretical guidance, all of which originated from foreign countries. Consequently, the adaptation of SDM theorical systems to local contexts is necessary in mainland China. The results of early attempts in this area have been encouraging. Zhang et al. (74) explored the process factors that influence SDM participation from the perspective of Chinese cancer patients and developed a conceptual framework for their involvement (103). The framework takes into consideration the special “filial culture” of China and focuses on the difficulties encountered by older adult patients in the decision support process. However, the framework has been published only recently and is limited in its target group, and is consequently in need of further validation.

### 4.3. More research is needed to focus on the decision-making needs of Chinese patients

Our review found that patient decision-makers face numerous challenges in SDM and need external support. These difficulties include three of the common types of decisional needs related to knowledge, decision aids, and aspects of self-efficacy. The three most common clinical manifestations were inadequate knowledge of the disease or condition to inform a decision (59%), lack of decision-support tools (43%), and low confidence in the ability to implement the chosen option (39%). Therefore, healthcare professionals should focus more on these aspects of the SDM process to promote patient involvement in decision-making.

Identifying patient decision needs is critical to facilitating patient participation in decision-making, as decision needs may be a determinant of sub-optimal decisions (104). Healthcare professionals can implement SDM by prioritizing screening for common manifestations of decision needs, which helps to quickly identify patients’ decision needs, implement shared decisions, and improve patient outcomes (31, 105, 106). In western countries, institutions represented by the University of Ottawa in Canada have conducted in-depth research on decision needs. They have clearly defined the concepts related to decision needs basing on the ODSF framework theory, so that medical practitioners can identify and assess the decision needs of patients and stakeholders in the decision-making process (107). Over 45 foreign studies have conducted decisional needs assessments based on the concept of decision needs in this theoretical framework (31), but we found no evidence from China. Even though research teams in the Chinese region have translated the concepts related to ODSF (108), they have not yet localized and debugged the relevant concepts to fit their own clinical context. The difficulty (rigidly understanding) in recognizing decision needs concepts by Chinese researchers and the gap in results from foreign studies (25) may be due to this difficulty. For example, “decision motivation” is a relatively low-frequency patient decision need in this study, which may be influenced by the traditional Chinese culture, where Chinese people are more introverted. In the long-term doctor-led decision-making model, patients are used to relying on doctors (54, 72, 86, 99) and passively participating in decision-making, making it difficult to detect decision motivation, which researchers often overlook. In addition, there are no uniform methodology standards for developing local and disease-appropriate decision needs survey tools/methods based on ODSF. Therefore, how to effectively define the concept of decision needs applicable to the Chinese cultural and medical scenario is a more pressing research problem and a key component in determining the successful implementation of shared decision making in the Chinese context. At the same time, to facilitate smooth SDM, it is necessary to understand further the common manifestations of decision needs faced by patient decision-makers in different decision areas, which will facilitate further understanding of the strategies used by Chinese patient decision-makers to cope with decision difficulties. These efforts have helped to explore the development of decision actively needs assessment tools or methods that are appropriate to the current state of local healthcare scenarios and populations and have helped to increase the chances that patient decision-makers will be supported in shared decision-making.

### 4.4. Shared decision-making workflow in mainland China needs further optimization

In this review, we developed initial codes based on the IP-SDM model (42). The model has the potential to improve decision-making processes and working practices currently exercised in many industrialized healthcare systems. The primary reason for the selection of the IP-SDM model was the chronological order of its constructs. By mapping the extracted contents to its constructs, we can identify the hotspots and developmental stages of Chinese SDM research. As depicted in Figure 2, Our analysis reveals that most Chinese SDM studies have focused on initiation, decision problems, information exchange, values, and preferences. In comparison, fewer studies have focused on factors such as feasibility, preferences, actual choices, implementation, and outcomes. The main reason for this finding may be that Chinese SDM research has just begun and is still in the tool development and localization process. Consequently, most studies may focus on the theoretical connotations of the SDM process, while fewer studies may address implementation (109). Such results provide our researchers with directions for future research. The successful implementation of SDM in China could present a substantial challenge.

Next, we can consider drawing on theories and methods from the field of implementation science to advance implementation research of SDM (110, 111). We found that physicians and nurses were the primary implementers of SDM in China. However, only a few studies mentioned other stakeholders, such as psychologists and volunteers. Such a status contrasts with the internationally promoted concept of multidisciplinary medical teams (112, 113). However, not all studies have proven that multidisciplinary medical teams yield better SDM outcomes; for example, patients may have difficulty in judging the most critical information after being presented with an overwhelming amount of factual detail. Furthermore, increased time costs are often required for multiple participants to reach a final agreement (114, 115). In a developing country such as China, where the doctor-patient ratio is meager, an emphasis on multidisciplinary team participation in SDM may not be an optimal choice. However, this issue is controversial and needs further discussion.

We also applied the Three Talk model to map the timing of the doctor-patient conversation. In the model, SDM is described in three key steps: team talk, option talk, and decision talk (43). The meanings of “build relationships” and “work together” used in SDM and the Three Talk model are substantially different. In the Chinese medical context, establishing relationships usually comprises the building of trust between the doctor and the patient (116). Western doctor-patient relationships are usually based on “institutional trust,” (117) but in China, multiple historical and cultural influences, professional ethics, medical and cultural institutions, and kinship-based ethical relationships are the general mechanisms for building trust in the medical experience (118, 119). Geographic ties, a sense of community membership, and local social opinions also play a significant role (120). Neither interpersonal nor purely institutional trust are established between doctors and patients; this process is culturally specific (121) and deserves to be explored in depth by SDM researchers. As shown in Figure 3, We also found new themes in the “post-decisional interview” “conversation on psychology and emotions.” Decision-making does not end with a decision but also requires attention to the patient’s short-term and long-term feedback (122). Post-decisional talk not only benefits the evaluation of SDM effectiveness, but also enhances the cultural adaptation of the talk model, which contributes to sustainable SDM application.

### 4.5. Research on patient decision aids in mainland China is in the developmental stage

Most studies that refer to decision support tools are unnamed, and less than half of the tools are designated as patient decision aids. The tools are currently presented in a variety of formats; half are paper-based materials. Less than 1/3 of the studies of support tools mentioned the process of tool development. Illustrated by Figure 4, We mapped our results to the National Institute for Health and Care Excellence framework for shared-decision-making support tools, and found that none of the studies described step 8: Supporting resources, step 10: Release and dissemination, and step 11: Update instructions. Only one study explicitly stated that it evaluated the tool using International Patient Decision Analysis Standards (IPDAS) guidance (9). Despite the proliferation of patient decision aids, information on their development processes is limited. Our finding that many of the patient decision aid trial reports failed to provide clear information regarding tool development was disappointing. Consequently, we systematically searched Chinese websites and found that the only Chinese article that systematically introduced specific IPDAS content was published in 2020 (123). Furthermore, no report on the production of a final version for use and/or further evaluation has been published by Chinese researchers. We can therefore conclude that the introduction and local adaptation of standards and development guidelines are urgently needed.

### 4.6. Shared decision-making outcome indicators in mainland China differ from those of western countries

The conceptual framework to guide analysis of outcome indicators was adapted from Street and colleagues’ and Kreps and colleagues’ (124, 125) models by Shay and Lafata (35). Shay and Lafata adapted models by Street and colleagues and Kreps and colleagues to build a conceptual framework to guide analysis of outcome indicators. Street and Kreps reported 68 outcomes, most of which were classified as affective-cognitive outcomes, followed by health outcomes. The fewest were classified as behavioral outcomes. In comparison, Shay and Lafata assessed 97 unique patient outcomes. Most were classified as affective-cognitive outcomes, followed by behavioral outcomes, and finally by health outcomes. Another study on personal health record technology showed the same trend as Shay and Lafata’s results (126). Referring to Figure 5, Our findings were congruent with these two SDM system evaluations in that most focused indicators belong to affective-cognitive outcomes. However, the focus on health outcomes has been higher in Chinese SDM studies than in the other two reports. Maslow classified human needs in a hierarchy that ascends through physiological, security, social, esteem, and self-actualization needs (127) Higher-level needs can only emerge after the previous lower-level needs are satisfied (127). The health outcomes of this study are physiological needs. Chinese research of SDM and also of the entire healthcare-related field generally focus more on health outcomes than western studies. This prioritization is necessitated by the large population size and the unbalanced and insufficient development and discrepant supply and demand of healthcare resources in China (128). The imbalance in healthcare resources is the major factor that necessitates the reporting of health outcomes in healthcare research in mainland China. This research can aid healthcare professionals in decision-making regarding the adoption or non-adoption of interventions to prioritize the basic needs of most patients in the context of limited resources (129).

Our results also showed that economic effects such as length of stay and costs could not be classified in Shay and Lafata’s model. An alternative outcome imputation model that considers all SDM consequences was proposed by Elwyn et al. (130) Their model includes proximal, distal, and distant effects; and considers the economic outcomes of SDM as distant effects because they take longer to manifest and are therefore less relevant as short-term metrics (130). Whether patient decision aids will generate savings is unclear. Greater consensus on what constitutes a patient decision aid and comparisons of their use to usual care over a sufficient timespan are required for the valid assessment of cost outcomes (131).

## 5. Strengths and limitations

The main strength of this integrated approach is that we can take a broader perspective when analyzing the current state of shared decision making implementation in the Chinese region. Understanding the current state of decision needs, decision support, and decision closure for shared decision making is critical to designing or developing more effective shared decision solutions for mainland China and facilitating effective implementation of SDM. For the decision needs analysis, the original studies we included were not initially based on the ODSF, so matching using ODSF decision needs codes may have been biased. In addition, due to the lack of perspectives on the decision-making needs of families supporting patient decision-makers and healthcare professionals makers in the included literature, we only analyzed the decision-making needs of decision-makers regarding patients. We did not explore the decision-making needs of families supporting patient decision-makers and healthcare professionals involved in SDM. This may have overlooked very important caregiver and healthcare worker-related information. In extracting the content of the articles, we did not refer to IP-SDM, Three-Talk, NICE guideline, Shay and Lafata’s model, which resulted in more work and less “linguistic” fit for the content analysis. However, this method of extraction also ensured that we obtained constructs that were not available in the above models, making our findings more informative. The results were presented and discussed in relation to the SDM implementation process, the timing of the conversation, and the tool development process without exploring the coverage of each study as an individual study, but with a broader perspective on the overall research process. This may have caused us to overlook some studies that are very representative of the current state of SDM in China. The minimal representation of Organizational and system level characteristics that influence the implementation of SDM in the full-text presentation may reflect the neglect of these factors in SDM research in China. However, it may also be that the extraction basis chosen for this study the theoretical framework ignored these factors, resulting in the omission of this study.

## 6. Conclusion

In this scoping review, we set out to map the findings of existing studies on SDM in mainland China. In terms of decisional needs, we found that patient decision-makers require more external support to overcome challenges in knowledge, decision assistance, and self-efficacy. However, the current lack of local definition of decisional needs will be detrimental to the development of SDM in mainland China. Therefore, it is necessary to conduct a follow-up study on the concept and expression of decisional needs in the Chinese cultural context. The review and summary of the implementation process of SDM and the construction process of PDAs in this study suggested that the scientific and systematic construction of PDAs should be paid attention to in the subsequent research process, and the combination of steps and context should be paid attention to in the implementation of SDM. Moreover, the focus on health outcomes in Chinese SDM studies, driven by the unique healthcare resource landscape, underscores the necessity of prioritizing basic needs within limited resources.

## Data availability statement

The original contributions presented in the study are included in the article/Supplementary material, further inquiries can be directed to the corresponding authors.

## Author contributions

XL, JZ, and YH: conceptualization. DY, MM, YY, XZ, HW, QL, and ML: data curation. XL and DY: formal analysis. DY, XL, and MM: methodology and writing-original draft. XL: project administration. JZ, YH, and JL: writing-review and editing. All authors contributed to the article and approved the submitted version.

## Funding

This paper was supported by basic Research Grant Program for 2023 at Beijing University of Chinese Medicine (project level; Grant Number 2023-JYB-XJSJJ019). The funders are not involved in the study design, data collection and analysis, manuscript writing or the decision to submit the manuscript for publication.

## Conflict of interest

The authors declare that the research was conducted in the absence of any commercial or financial relationships that could be construed as a potential conflict of interest.

## Publisher’s note

All claims expressed in this article are solely those of the authors and do not necessarily represent those of their affiliated organizations, or those of the publisher, the editors and the reviewers. Any product that may be evaluated in this article, or claim that may be made by its manufacturer, is not guaranteed or endorsed by the publisher.

## Footnotes

^1^ https://xueshu.baidu.com/
