# Supplementary material for: Shared decision-making in healthcare in mainland China: a scoping review
Source: Front Public Health. 2023 Sep 7;11:1162993. doi: 10.3389/fpubh.2023.1162993 (PMC10513465; doi:10.3389/fpubh.2023.1162993)
Supplement: Supplementary file 2 [file Table_2.DOCX]

**Appendix A.** Search strategies

| **Database** | **Search strategy** | |
| --- | --- | --- |
| **Pubmed**  Limits: 1968/1/1 to 2021/4/30 | #1 | ((("Decision Making"[MeSH]) OR ("Decision Making, Shared"[MeSH])) OR ("Decision Making, Shared"[Title/Abstract])) OR ("Decision Making"[Title/Abstract]) |
|  | #2 | ((((collaborative[Title/Abstract]) OR (shared[Title/Abstract])) OR (informed[Title/Abstract])) OR (cooperative[Title/Abstract])) AND ("decision making"[Title/Abstract]) |
|  | #3 | ((((involved[Title/Abstract]) OR (involvement[Title/Abstract])) OR ("Patient Preference"[Title/Abstract])) OR ("patient preference"[MeSH])) AND ("decision making"[Title/Abstract]) |
|  | #4 | #1 OR #2 OR #3 |
|  | #5 | (China [Mesh]) |
|  | #6 | #4 AND #5 |
| **EMBASE**  1968/1/1 to 2021/4/30 | #1 | ('decision making'/exp OR 'decision making, shared'/exp OR 'decision making':ti,ab,kw OR 'decision making, shared':ti,ab,kw) AND [1968-2021]/py |
|  | #2 | (shared:ti,ab,kw OR informed:ti,ab,kw OR cooperative:ti,ab,kw OR collaborative:ti,ab,kw) AND 'decision making':ti,ab,kw AND [1968-2021]/py |
|  | #3 | (involved:ti,ab,kw OR involvement:ti,ab,kw OR 'patient preference'/exp OR 'patient preference':ti,ab,kw) AND 'decision making':ti,ab,kw |
|  | #4 | #1 OR #2 OR #3 |
|  | #5 | 'china'/exp |
|  | #6 | #4 AND #5 |
| **CINAHL**  Limits: 1968/1/1 to 2021/4/30 | #1 | TI "Decision Making" OR TI "Decision Making, Shared" |
|  | #2 | TI ( Shared OR Informed OR Cooperative OR Collaborative ) AND TI "decision making" |
|  | #3 | TI ( Involved OR Involvement OR "patient Preference" ) AND TI "decision making" |
|  | #4 | #1 OR #2 OR #3 |
|  | #5 | TI China |
|  | #6 | #4 AND #5 |
| **PsycINFO**  Limits: 1968/1/1 to 2021/4/30 | #1 | TI "Decision Making" OR TI "Decision Making, Shared" |
|  | #2 | TI ( Shared OR Informed OR Cooperative OR Collaborative ) AND TI "decision making" |
|  | #3 | TI ( Involved OR Involvement OR "patient Preference" ) AND TI "decision making" |
|  | #4 | #1 OR #2 OR #3 |
|  | #5 | TI China |
|  | #6 | #4 AND #5 |
| **Web of Science**  Limits: 1968/1/1 to 2021/4/30 | #1 | TI=("Decision Making" OR "Decision Making, Shared") |
|  | #2 | TI=(Shared OR Informed OR Cooperative OR Collaborative ) AND TI=("decision making") |
|  | #3 | TI=(Involved OR Involvement OR "patient Preference") AND TI=("decision making") |
|  | #4 | #1 OR #2 OR #3 |
|  | #5 | TI=(China) |
|  | #6 | #4 AND #5 |
| **Cochrane Library** | #1 | MeSH descriptor: [Decision Making] explode all trees |
|  | #2 | MeSH descriptor: [Decision Making, Shared] explode all trees |
|  | #3 | ("Decision Making" OR "Decision Making, Shared"):ti,ab,kw |
|  | #4 | #1 OR #2 OR #3 |
|  | #5 | (Shared OR Informed OR Cooperative OR Collaborative):ti,ab,kw AND ("decision making"):ti,ab,kw |
|  | #6 | MeSH descriptor: [Patient Preference] explode all trees |
|  | #7 | (Involved OR Involvement OR "patient Preference"):ti,ab,kw |
|  | #8 | #6 OR #7 |
|  | #9 | (China):ti,ab,kw |
|  | #10 | #4 OR #5 OR #8 |
|  | #11 | #9 AND #10 with Cochrane Library publication date Between Jan 1968 and Apr 2021, in Cochrane Reviews, Cochrane Protocols, Trials, Clinical Answers, Editorials, Special Collections |
| **CNKI**  Limits: 1968/1/1 to 2021/4/30 | #1 | (篇名=共同决策 + 共享决策 + 决策辅助 + 决策工具 + 决策支持 + 决策参与 + 医疗决策 + 医患决策) |
| **WanFang**  Limits: 1968/1/1 to 2021/4/30 | #1 | 题名或关键词:(共同决策 OR 共享决策 OR 决策辅助 OR 决策工具 OR 决策支持 OR 决策参与 OR 医疗决策 OR 医患决策) |
| **VIP**  Limits: 1968/1/1 to 2021/4/30 | #1 | 题名=共同决策 OR 共享决策 OR 决策辅助 OR 决策工具 OR 决策支持 OR 决策参与 OR 医疗决策 OR 医患决策 |
| **CBM**  Limits: 1968/1/1 to 2021/4/30 | #1 | ( "共同决策"[标题:智能] OR "共享决策"[标题:智能] OR "决策辅助"[标题:智能] OR "决策工具"[标题:智能] OR "决策支持"[标题:智能] OR "决策参与"[标题:智能] OR "医疗决策"[标题:智能] OR "医患决策"[标题:智能]) |
