# Supplementary material for: Shared decision-making in healthcare in mainland China: a scoping review
Source: Front Public Health. 2023 Sep 7;11:1162993. doi: 10.3389/fpubh.2023.1162993 (PMC10513465; doi:10.3389/fpubh.2023.1162993)
Supplement: Supplementary file 3 [file Table_3.DOCX]

**Additional file 2 Summary of the characteristics of the included studies (n = 60).**

| **Authors/ Year** | **Purpose/Aim** | **Study Design** | **research area** | **Decisional needs**  **(attached documents)** | **Decision outcome** | **Decision support** | |
| --- | --- | --- | --- | --- | --- | --- | --- |
|  |  |  |  |  |  | **name** | **type** |
| **Zhao et al**  **(2018)**[1] | **(1) By combing the history of the concept of "patient participation", this paper expounds on the meaning of "patient participation" in clinical decision-making and discusses its theoretical and practical significance from the perspective of obligation theory and utility theory;**  **(2) Analyze the main practical ethical problems faced by "patient participation" in China.** | **Cross-sectional study** | **Outpatients and inpatients** | **1. Unrealistic Expectations**  **2. Unclear Values** | **None** | **None** | **None** |
| **Zhang et al**  **(2010)**[2] | **To understand the factors affecting doctor-patient shared clinical decision-making and to provide ideas and Countermeasures for doctor-patient shared clinical decision-making.** | **Cross-sectional study** | **Patients in the outpatient department and inpatient department** | **1. Decisional conflict**  **2. Inadequate Knowledge** | **None** | **None** | **None** |
| **Zhang et al.**  **(2017)**[3] | **To understand the current situation and influencing factors of satisfaction of malignant tumor patients participating in medical and nursing decision-making to provide a reference basis for improving the quality of medical and nursing services in the future.** | **Cross-sectional study** | **Malignant tumor patients** | 1. **Inadequate Knowledge** 2. **Inadequate self-efficacy** 3. **Inadequate information** 4. **Inadequate advice** 5. **Personal Needs** | **None** | **None** | **None** |
| **Guo et al.**  **(2020)**[4] | **To understand the current situation of treatment scheme sharing decision-making in patients with rectal cancer undergoing enterostomy, and to understand the true feelings of patients who were told or concealed the operation scheme, to provide the basis for making a more beneficial sharing decision-making scheme for patients.** | **Qualitative study** | **Patients with rectal cancer undergoing enterostomy** | 1. **Inadequate Knowledge** 2. **Unclear Values** 3. **Social Pressure** 4. **Inadequate advice** 5. **Inadequate emotional support** 6. **Inadequate health and social services** 7. **Clinical Needs** | **None** | **None** | **None** |
| **Zheng (2019)**[5] | **(1) Through qualitative research, understand the attitudes and influencing factors of medical staff, maternal and family members to participate in childbirth decision-making jointly, encourage maternal and family members to participate in the choice of childbirth mode jointly, select the most suitable childbirth mode, prevent and reduce childbirth**  **(2) To understand the influencing factors of maternal delivery mode selection, provide the scientific basis for medical staff to implement standardized and personalized health guidance, and provide the basis for the smooth development of clinical decision-making.** | **Mixed research** | **(cesarean section / vaginal) delivery** | 1. **Inadequate Knowledge** 2. **Inadequate self-efficacy** 3. **Inadequate advice** 4. **Inadequate emotional support** | **None** | **None** | **None** |
| **Ming et al**  **(2018)**[6] | **To study the patient participation in decision-making in the clinical application of new medical technology in China and analyze the correlation and impact of patient participation in decision-making on the use behavior of new medical technology.** | **Cross-sectional study** | **Patients involved in shared decision-making** |  | **None** | **None** |  |
| **Yuan et al**  **(2014)**[7] | **To understand the willingness of general surgical patients to participate in surgical decision-making and its influencing factors to provide the basis for the implementation of doctor-patient surgical decision-making.** | **Cross-sectional study** | **General surgical patients** | 1. **Decisional conflict** 2. **Inadequate Knowledge** 3. **Inadequate information** 4. **Inadequate financial assistance** | **None** | **None** |  |
| **Zhang et al**  **(2020)**[8] | **To understand the influencing factors and process of cancer patients' participation in clinical decision-making and construct the conceptual framework of cancer patients&apos; participation in the shared decision-making process.** | **Cross-sectional study** | **Cancer patients** | 1. **Inadequate Knowledge** 2. **Social Pressure** 3. **Difficult decisional roles** 4. **Inadequate emotional support** 5. **Inadequate health and social services** | **None** | **None** | **None** |
| **Li (2019)**[9] | **To investigate the current status and influencing factors of thyroid cancer patients' participation in treatment decision-making. To understand the attitude of patients and their families towards patients and its influencing factors.**  **To understand the attitude of traditional Chinese medicine (TCM) students towards patients and its influencing factors. To understand the participation in treatment decision-making in the process of treatment, so as to provide theoretical basis for the construction of treatment decision-making model for thyroid cancer patients.** | **Mixed research** | **Thyroid cancer patients** | 1. **Inadequate Knowledge** 2. **Inadequate experience** 3. **Inadequate self-efficacy** 4. **Inadequate financial assistance** 5. **Personal Needs** | **None** | **None** | **None** |
| **Dian et al**  **(2019)**[10] | **To understand the experience of elderly patients with macular degeneration in the decision-making process of intravitreal injection of anti-neovascular drugs to provide a theoretical basis for guiding patients to effectively participate in joint decision-making and improve the quality of decision-making.** | **Qualitative study** | **Elderly patients with macular degeneration** | 1. **Decisional conflict** 2. **Inadequate Knowledge** 3. **Social Pressure** 4. **Inadequate advice** 5. **Difficult Decision Timing** | **None** | **None** | **None** |
| **Zheng et al**  **(2020)**[11] | **Describe the obstacles and promoting factors of shared decision-making to provide a basis for further clinical implementation of shared decision-making.** | **Qualitative study** | **Breast cancer patients** | 1. **Decisional conflict** 2. **Unclear Values** 3. **Difficult decisional roles** 4. **Inadequate self-efficacy** 5. **Inadequate health and social services** 6. **Difficult Decision Timing** 7. **Personal Needs** | **None** | **None** | **None** |
| **Fu et al**  **(2016)**[12] | **The objective is to investigate the needs of breast cancer patients with different characteristics to participate in medical decision-making.** | **Cross-sectional study** | **Breast cancer patients** | 1. **Inadequate Knowledge** 2. **Inadequate financial assistance** 3. **Personal Needs** | **None** | **None** | **None** |
| **Cai et al**  **(2020)**[13] | **To understand the true feelings of breast cancer patients participating in treatment and nursing decision-making and the difficulties encountered by patients in the decision-making process to provide the basis for making targeted decision-making.** | **Qualitative study** | **Breast cancer patients** | 1. **Decisional conflict** 2. **Inadequate Knowledge** 3. **Difficult decisional roles** 4. **Inadequate self-efficacy** 5. **Inadequate information** 6. **Inadequate emotional support** | **None** | **None** | **None** |
| **Wan et al**  **(2020)**[14] | **To understand the influencing factors of joint decision-making in patients with wet age-related macular degeneration.** | **Qualitative study** | **Patients with wet age-related macular degeneration** | 1. **Inadequate Knowledge** 2. **Unrealistic Expectations** 3. **Difficult decisional roles** 4. **Inadequate self-efficacy** 5. **Inadequate information** 6. **Inadequate instrumental help** 7. **Inadequate health and social services** 8. **Difficult Decision Timing** 9. **Personal Needs** | **None** | **None** | **None** |
| **Zhang et al**  **(2013)**[15] | **Combined with the research on the doctor-patient relationship, this paper analyzes the possible causes of the tension between doctors and patients. It puts forward that doctors and patients should actively encourage patients to participate in clinical decision-making to find an effective way to alleviate the tension between doctors and patients.** | **Cross-sectional study** | **Outpatients and inpatients** | 1. **Decisional conflict** 2. **Inadequate Knowledge** 3. **Inadequate information** | **None** | **None** | **None** |
| **Feng et al**  **(2021)**[16] | **The objective is to understand the attitude of diabetes patients toward using decision-support tools to help them participate in doctor-patient decision-making.** | **Cross-sectional study** | **Diabetic** | 1. **Inadequate Knowledge** 2. **Inadequate experience** 3. **Inadequate motivation** 4. **Inadequate information** 5. **Difficult Decision Type** | **None** | **None** | **None** |
| **Yuan et al**  **(2017)**[17] | **To investigate the current situation of surgical patients&apos; actual participation in surgical decision-making and analyze the relevant influencing factors.** | **Cross-sectional study** | **Patients after surgery** | 1. **Inadequate Knowledge** 2. **Social Pressure** 3. **Difficult decisional roles** 4. **Inadequate self-efficacy** 5. **Inadequate motivation** 6. **Inadequate emotional support** 7. **Inadequate financial assistance** | **None** | **None** | **None** |
| **Zhang et al**  **(2020)**[18] | **To understand the experience of patients with advanced lung cancer participating in and implementing treatment decision-making, and understand the supportive needs of patients in the treatment process, to provide a reference for the construction of a treatment decision-making support scheme led by specialist nurses.** | **Qualitative study** | **Patients with advanced lung cancer** | 1. **Inadequate Knowledge** 2. **Inadequate self-efficacy** 3. **Inadequate emotional support** 4. **Inadequate financial assistance** | **None** | **None** | **None** |
| **Wang et al**  **(2021)**[19] | **To study the participation of patients with chronic diseases in drug decision-making and understand its influencing factors and internal mechanism to provide a reference for promoting a doctor-patient joint decision-making model.** | **Cross-sectional study** | **Patients with chronic diseases** | 1. **Inadequate Knowledge** 2. **Difficult Decision Timing** | **None** | **None** | **None** |
| **Xie et al**  **(2020)**[20] | **To understand the conflicting dilemma and influencing factors of patients with atrial fibrillation participating in anticoagulation treatment decision-making to provide the basis for understanding their experience of participating in decision-making and making personalized decision-making assistance schemes.** | **Qualitative study** | **Patients with atrial fibrillation** | 1. **Decisional conflict** 2. **Difficult decisional roles** 3. **Inadequate information** 4. **Inadequate emotional support** 5. **Inadequate emotional support** | **None** | **None** | **None** |
| **Liu et al**  **(2021)**[21] | **To understand the current expectation, ability, and satisfaction of patients with inflammatory bowel disease participating in clinical decision-making, and analyze the relationship between them, to provide a reference for improving patients&apos; satisfaction in clinical decision-making.** | **Cross-sectional study** | **Patients with inflammatory bowel disease** | 1. **Inadequate Knowledge** 2. **Inadequate self-efficacy** 3. **Inadequate advice** 4. **Inadequate health and social services** | **None** | **None** | **None** |
| **Su et al**  **(2020)**[22] | **To understand the treatment decision dilemma of ophthalmectomy patients** | **Qualitative study** | **Patients with enucleation** | 1. **lacks the ability or skill to make a decision** 2. **Inadequate emotional support** 3. **Inadequate instrumental help** | **None** | **None** | **None** |
| **Yang (2014)**[23] | **To** **understand the related factors affecting the willingness of schizophrenic patients to participate in medical decision-making.** | **Cross-sectional study** | **Schizophrenic patients** | 1. **Inadequate experience.**   **2. Inadequate financial assistance** | **None** | **None** | **None** |
| **Bai et al**  **(2017)**[24] | **To understand the expectation of patients with advanced cancer to participate in clinical decision-making to provide better medical and nursing services for patients.** | **Cross-sectional study** | **Patients with advanced tumor** | 1. **Inadequate Knowledge**   **2.Inadequate Knowledge** | **None** | **None** | **None** |
| **Huang et al**  **(2020)**[25] | **To understand joint decision-making from the perspective of hospitalized schizophrenics in China.** | **Qualitative study** | **Schizophrenic patients** | 1. **Decisional conflict** 2. **Difficult decisional roles** 3. **Inadequate self-efficacy** 4. **lacks the ability or skill to make a decision** 5. **Inadequate advice** 6. **Inadequate emotional support** 7. **Inadequate health and social services** 8. **Inadequate financial assistance** 9. **Difficult Decision Timing** 10. **Personal Needs** | **None** | **None** | **None** |
| **Huang et al**  **(2019)**[26] | **Compare the decision-making preferences of patients and mental health experts in China and Europe.** | **Cross-sectional study** | **Patients with severe mental illness, MHPs (mental health professionals)** | 1. **Inadequate Knowledge** 2. **Difficult decisional roles** 3. **Inadequate self-efficacy** 4. **lacks the ability or skill to make a decision** 5. **Inadequate information** 6. **Personal Needs** | **None** | **None** | **None** |
| **Han et al**  **(2021)**[27] | **The objective is to understand the surgical decision-making conflicts and related factors in early breast cancer patients in China.** | **Cross-sectional study** | **Patients with early breast cancer face surgical decisions** | 1. **Decisional conflict** 2. **Inadequate Knowledge** 3. **Difficult decisional roles** 4. **Inadequate self-efficacy** 5. **Inadequate emotional support**   **Difficult**   1. **Decision Timing** | **None** | **None** | **None** |
| **Luo et al (2021)**[28] | **To understand the SDM of inpatients in tertiary public hospitals in Shanghai and the influencing factors of SDM, and to determine whether SDM will lead to higher satisfaction with inpatient care, medical services, medical expenses, and treatment results.** | **Cross-sectional study** | **Inpatients in tertiary public hospitals** | 1. **Inadequate experience** 2. **Inadequate information**   **Difficult**   1. **Decision Type** | **None** | **None** | **None** |
| **Wu (2019)**[29] | **The purpose of this study is to understand the factors that affect AF patients' participation in decision-making, objectively analyze the characteristics of decision-making information processing, provide a basis for subsequent intervention programs, promote AF patients' participation in treatment decision-making, and provide guidance for clinical treatment decision-making communication.** | **Mixed research** | **Patients with atrial fibrillation** |  | **None** | **None** | **None** |
| **Wu et al (2019)**[30] | **In order to understand the application value of the "joint decision-making model" in ICU doctor-patient communication** | **NRCT** | **ICU critically ill patients** | 1. **Difficult decisional roles** 2. **Inadequate instrumental help** | **ICU hospitalization expenses, ICU length of stay, anxiety and depression scores of patients.** | **Not specified** | **Local Chinese version** |
| **Ye et al. (2021)**[31] | **Design and implement PICC informed consent mobile medical decision-making assistance program to enhance patients&apos; willingness to participate in decision-making.** | **RCT** | **Gynecological tumor patients** | 1. **Decisional conflict** 2. **Inadequate emotional support** 3. **Inadequate instrumental help** | **Anxiety and depression scores, and patient satisfaction scores.** | **PICC patient informed consent decision aid kit** | **Local Chinese version** |
| **Yang (2019)**[32] | **The objective is to understand the effect of CO decision mode in the functional exercise of postoperative breast cancer patients.** | **NRCT** | **Postoperative breast cancer patients (limb function) [functional exercise]** | 1. **Inadequate Knowledge** 2. **Inadequate self-efficacy** 3. **Inadequate instrumental help** 4. **Clinical Needs** | **Limb dysfunction score, functional exercise compliance score, and quality of life score.** | **Video, manual, poster, role play, Internet and other decision-making aids** | **Local Chinese version** |
| **Wu (2020)**[33] | **To understand the impact of shared decision-making (SDM) intervention on patients with coronary heart disease to improve decision-making participation and decision-making satisfaction of patients with coronary heart disease and reduce doctor-patient decision-making conflict.** | **NRCT** | **Patient with coronary heart disease** | 1. **Inadequate instrumental help** 2. **Inadequate Knowledge** 3. **Inadequate self-efficacy** 4. **Inadequate instrumental help** 5. **Clinical Needs** | **None** | **Coronary heart disease SDM dry forecast** | **Local Chinese version** |
| **Wu et al**  **(2019)**[34] | **To understand the impact of the shared decision-making model on decision-making intention and condition management of patients with cerebral infarction.** | **RCT** | **Patients with cerebral infarction** | 1. **Inadequate Knowledge** 2. **Inadequate self-efficacy** 3. **Inadequate instrumental help** | **None** | **Nursing system** | **Local Chinese version** |
| **Zhang et al**  **(2019)**[35] | **To understand the value of shared decision-making nursing in rapid rehabilitation of knee arthroplasty.** | **NRCT** | **Patients undergoing joint replacement surgery (perioperative period)** | 1. **Decisional conflict** 2. **Inadequate Knowledge** 3. **Unrealistic Expectations** 4. **Inadequate emotional support** 5. **Clinical Needs** 6. **Inadequate instrumental help** | **Anxiety score, knee flexion Angle, pain score, length of hospital stay, and hospitalization cost.** | **nothing** | **Local Chinese version** |
| **Qian et al**  **(2020)**[36] | **To observe the effect of shared decision-making mode on completeness of chemotherapy and satisfaction with decision-making in breast cancer patients.** | **NRCT** | **Breast cancer patients undergoing chemotherapy** | 1. **Inadequate Knowledge** 2. **Inadequate self-efficacy** 3. **Inadequate self-efficacy** | **Completion rate of chemotherapy and satisfaction of decision-making participation.** | **Health decision support manual** | **Local Chinese version** |
| **Xie (2021)**[37] | **To understand the application and effect of the doctor-patient sharing decision-making model in emergency patients with hypertension.** | **RCT** | **Emergency hypertensive patients** | 1. **Inadequate instrumental help** 2. **Personal Needs** | **Effective blood pressure control time, average emergency treatment time, and patient satisfaction score.** | **Sharing decision-making and treatment plan between doctors and patients** | **Local Chinese version** |
| **Du (2021)**[38] | **To analyze the effect of shared decision-making rapid rehabilitation nursing on anxiety and joint function recovery of patients with knee arthroplasty.** | **RCT** | **Patients with knee arthroplasty** | 1. **Decisional conflict** 2. **Inadequate Knowledge** 3. **Inadequate emotional support** 4. **Inadequate instrumental help** 5. **Clinical Needs** | **Self-rating anxiety scale, joint function recovery rate** | **nothing** | **Local Chinese version** |
| **Liu et al**  **(2020)** [39] | **To understand the role of shared decision-making in promoting the rapid rehabilitation of children with congenital pseudarthrosis of the tibia.** | **NRCT** | **Patients with congenital pseudarthrosis of tibia** | 1. **Inadequate emotional support** 2. **Inadequate instrumental help** 3. **Inadequate financial assistance** 4. **Clinical Needs** | **Anxiety score, pain score, length of stay, hospitalization cost, patient satisfaction.** | **Rapid rehabilitation program** | **Local Chinese version** |
| **Shi et al. (2019)**[40] | **To construct a decision aid for Chinese patients with implantable cardioverter defibrillator (ICD), and to provide solutions for medical decision-making of such patients, so as to promote the development of shared decision-making between doctors and patients.** | **Mixed research** | **Patients with implantable cardioverter defibrillator** | 1. **Inadequate instrumental help** | **The acceptance and attitude of patients and their families to the tool** | **Decision aid for patients with implantable cardioverter defibrillator** | **Local Chinese version** |
| **Liu et al.**  **(2021)**[41] | **To understand the effect of medical care family interaction intervention based on shared decision-making on patients with gastric cancer.** | **RCT** | **Gastric cancer patients** | 1. **Inadequate instrumental help** | **Self-rating anxiety scale (SAS), self-rating depression scale (SDS), human serum albumin (ALB), prealbumin (pre), transferrin (TRF), anal exhaust time, gastrointestinal function recovery time, ambulation time, length of hospital stay, incidence of complications.** | **Information sharing platform** | **Local Chinese version** |
| **Mou et al.**  **(2019)**[42] | **To analyze the impact of the patient participation decision-making model based on nurse-patient harmony on decision-making intention and condition management of patients in the neurology department.** | **NRCT** | **Neurology patients** | 1. **Inadequate Knowledge** 2. **Inadequate self-efficacy** 3. **Inadequate instrumental help** 4. **Clinical Needs** | **None** | **nothing** | **Local Chinese version** |
| **Li et al. (2019)**[43] | **The objective is to evaluate the effect of individualized management based on doctor-patient decision-making (SDM) for glycemic control and body mass index (BMI), blood pressure, blood lipids, self-management behaviors, and satisfaction with medical decision-making in patients with type 2 diabetes mellitus (T2DM) receiving insulin treatment.** | **RCT** | **Type 2 diabetic patients receiving insulin therapy** | 1. **Inadequate information** 2. **Inadequate instrumental help** | **FPG, 2hPG, HbA1c levels, exercise, blood glucose monitoring scores, treatment compliance scores, and satisfaction with decision-making participation scores.** | **Decision support manual for individualized management scheme of T2DM patients** | **Local Chinese version** |
| **Deng et al. (2019)**[44] | **The objective is to understand the value of the Decision-aid strategy in improving the compliance of diabetic patients and the primary prevention or two-level prevention of AS-CVD in people with diabetes.** | **RCT** | **Diabetic patients with atherosclerotic cardiovascular disease** | 1. **Inadequate Knowledge** 2. **Inadequate information** 3. **Inadequate instrumental help** | **The levels of TC, TG and LDL-C in patients, The compliance rate of the decision aid group, the satisfaction rate of patients, and the compliance score of the decision aid group** | **nothing** | **Local Chinese version** |
| **Zhou (2020)**[45] | **To understand the impact of shared decision perception intervention on health behavior and shared decision perception of patients with coronary heart disease under the guidance of goal control theory.** | **RCT** | **Patient with coronary heart disease** | 1. **Inadequate instrumental help** | **MLHFQ score, MLHFQ score, srahp score, mmas-8 score and sdm-q-9 score were scored.** | **nothing** | **Local Chinese version** |
| **Zhang et al.**  **(2020)**[46] | **To understand the effect of the rapid rehabilitation surgery (ERAS) model under doctor-nurse patient sharing decision-making in the perioperative period of esophageal cancer patients.** | **NRCT** | **Patients with esophageal cancer (perioperative period)** | 1. **Inadequate emotional support** 2. **Inadequate instrumental help** 3. **Clinical Needs** | **The recovery time of postoperative bowel sound, the time of first anal exhaust, the time of first oral feeding, the time of first ambulation, the length of postoperative hospital stay, the cost of hospitalization, and the incidence of adverse reactions were recorded.** | **Patient decision aids and decision coaching** |  |
| **Chen et al.**  **(2021)**[47] | **To understand the application effect of the doctor-patient joint decision-making model in acne patients.** | **RCT** | **Acne patients** | 1. **Inadequate Knowledge** 2. **Inadequate self-efficacy** 3. **Inadequate emotional support** 4. **Inadequate instrumental help** | **Self-rating Anxiety Scale (SAS), Self-rating Depression Scale (SDS), patient compliance behavior score, self-care knowledge level, treatment efficiency, skin lesion score.** | **nothing** | **Local Chinese version** |
| **Wang (2019)**[48] | **The objective is to understand the prognosis of patients with diabetes combined with cerebral infarction.** | **RCT** | **Diabetic patients with cerebral infarction** |  | **In the SDM group, plasma oxidative stress index, NIHSS score and glycosylated hemoglobin target rate were measured.** | **nothing** |  |
| **Ou et al. (2021)**[49] | **To understand the effect of the doctor-patient joint decision-making (SDM) intervention model in patients with the two-way affective disorder (BD).** | **NRCT** | **Patients with bipolar disorder** | 1. **Inadequate Knowledge** 2. **Inadequate information** | **WMS-Ⅳ, WISC-Ⅳ score and self-care Agency Scale score.** | **nothing** |  |
| **Li et al. (2020)**[50] | **To understand the effect of doctor-patient joint decision-making intervention in schizophrenic patients with persistent auditory hallucinations.** | **RCT** | **Schizophrenic patients with persistent auditory hallucinations** | 1. **Inadequate Knowledge** | **Positive symptoms, negative symptoms, auditory hallucinations, general psychopathology scores and total scores.** | **nothing** |  |
| **Chen et al**  **(2020)**[51] | **To understand the effect of doctor-patient joint decision-making (SDM) in preventing postoperative thrombosis in elderly patients with lower limb fractures.** | **NRCT** | **Elderly patients with lower limb fracture** | 1. **Inadequate Knowledge** 2. **Inadequate instrumental help** | **Health knowledge score, group compliance rate, anxiety, depression evaluation, nursing satisfaction score.** | **nothing** |  |
| **Liang et al**  **(2015)**[52] | **To understand the impact of the implementation of doctor-patient joint decision-making diagnosis and treatment model on the treatment compliance of schizophrenic patients.** | **RCT** | **Schizophrenic patients** | 1. **Inadequate self-efficacy** 2. **Inadequate Knowledge** | **BPRS score and treatment compliance score.** | **nothing** |  |
| **Zhao et al.**  **(2017)**[53] | **To understand the effect of doctor-patient joint decision-making diagnosis and treatment model on medication compliance and clinical efficacy of patients with mental disorders in the community.** | **RCT** | **Convalescent patients with mental disorders** |  | **Medication compliance score rate, BPRS, SDSS score.** | **nothing** |  |
| **Ding (2020)**[54] | **This study understand the nursing effect of the SDM model on HD patients.** | **NRCT** | **Chronic renal failure** | 1. **Inadequate self-efficacy** 2. **Inadequate emotional support** 3. **Clinical Needs** | **Patients were rated for positive attitudes towards reality and future, positive actions taken, and maintaining close relationships with others. Self-care skills, concepts, responsibilities, health knowledge, and self-care scores. Patient's degree of fatigue. Patients' quality of life score and the incidence of complications.** | **nothing** |  |
| **Meng et al.**  **(2018)**[55] | **To understand the role of treatment decision-making assistance in improving patients&apos; decision-making difficulties, anxiety, and depression.** | **NRCT** | **Lung cancer patients** | 1. **Inadequate emotional support** | **Patient decision distress score, hospitalization anxiety and depression score.** | **nothing** |  |
| **Guo (2020)**[56] | **To construct a decision-making assistant scheme for functional exercise in patients with phobia after unilateral total knee arthroplasty.** | **NRCT** | **Patients with unilateral total knee arthroplasty** | 1. **Decisional conflict** 2. **Inadequate Knowledge** 3. **Inadequate self-efficacy** 4. **Inadequate emotional support** 5. **Clinical Needs** | **The patients' quality of life, postoperative functional exercise compliance, knee joint function score, anxiety and depression score, decision-making satisfaction score and decision-making participation satisfaction score were scored.** | **nothing** |  |
| **Li (2017)**[57] | **To explore the role of shared decision-making model in the treatment of early PLC patients.**  **To understand how patients feel about being involved in treatment decisions.** | **Mixed research** | **Patients with early primary liver cancer** | 1. **Decisional conflict** 2. **Inadequate Knowledge** 3. **Inadequate self-efficacy** 4. **Inadequate self-efficacy** 5. **Inadequate emotional support** 6. **Inadequate instrumental help** 7. **Inadequate health and social services** 8. **Personal Needs** | **Patients' participation in treatment decision-making, anxiety and depression score, decision-making readiness score, decision-making distress score, and patient satisfaction.** | **Early PLC patient treatment decision aid kit** | **Local Chinese version** |
| **Shi (2019)**[58] | **To verify the intervention effect of decision-making aids for ICD patients on improving decision-making conflict.** | **RCT** | **Patients with implantable cardioverter defibrillator [patients at high risk of sudden cardiac death, ICD patients to be implanted and their families]** | 1. **Inadequate Knowledge** 2. **Inadequate instrumental help** 3. **Inadequate health and social services** | **Decision conflict score; Level of knowledge.** |  | **Local Chinese version** |
| **Wang et al.**  **(2021)**[59] | **To develop a mobile technology-based treatment decision-making aid platform for patients with primary liver cancer, so as to help patients understand the treatment information of liver cancer, improve the efficiency of doctor-patient communication, and improve the quality of shared decision-making.** | **Mixed research** | **Primary liver cancer** |  | **Patient compliance score；** | **PLC patient treatment decision support platform** | **Local Chinese version** |
| **Huang et al.**  **(2016)**[60] | **To evaluate the feasibility of using statins to assist decision-making.** | **Prospective, feasibility study** | **Patient with coronary heart disease** | **Inadequate instrumental help** | **Increased compliance;** | **Decision aid in statin selection** | **Adapted Edition** |

**NRCT:[Non-](http://www.baidu.com/link?url=0gj2Ta7C58alhhrurWUzp6PEtejqRihbvjiGhNwvxP1WoT7wD_W9m1xDj8i2bnK2-pZalLjgUFn-Ix8eA5FjtaLki2HhiStBDZvZclwcrzdt6Won1OZYpA5BVnXTMwpcI-AtFToM3dwVizyTfjbp1q)****[randomized control trial](http://www.baidu.com/link?url=0gj2Ta7C58alhhrurWUzp6PEtejqRihbvjiGhNwvxP1WoT7wD_W9m1xDj8i2bnK2-pZalLjgUFn-Ix8eA5FjtaLki2HhiStBDZvZclwcrzdt6Won1OZYpA5BVnXTMwpcI-AtFToM3dwVizyTfjbp1q);RCT:randomized control trial**

**References**

[1] Zhao Y, Zhang Q, Liang LZ. Study on the Theory and Practical Problem of“Patient Participation”in Clinical Decision Making. Chinese Medical Ethics 2018;31(06):799-803 (in Chinese).

[2] Zhang QW, Wan XL, Liu Y, et al. A Survey Analysis of Patients’Perceptions of Difficulties in Shared Clinical Decision-Making. Chinese Journal of Practical Nursing 2010;10(01):10-3 (in Chinese).

[3] Zhang HW, Hou XT, Bai DL, et al. The patients satisfaction with participation in medical and nursing decision making among cancer patients: a cross-sectional study. BMC Med Inform Decis Mak 2017;33(23):1805-9 (in Chinese).

[4] Guo Y, Wen XL, Xin X, et al. Inform Consent or Conceal the Truth: A Qualitative Study on the Real Experience of Sharing Decision-making between Doctors and Patients in Rectal Cancer Patients Undergoing Enterostomy. Chinese Medical Ethics 2020;33(01):80-4 (in Chinese).

[5] Zheng HX. Multi-dimensional research on the influence of maternal delivery decision from the perspective of shared decision making. 2019 (in Chinese).

[6] Ming J, Wei Y, He LY, et al. Corelation Analysis of Physician-Patient Shared Decision-making and the Adoption of New Medical Technologies. Chinese Hospital Management 2018;38(03):19-22 (in Chinese).

[7] Yuan YJ, Wu Y, Yan MQ. Study on the patients’preferences for involvement in surgical decision-making and influencing factors. Journal of Nursing Science 2014;29(10):23-5 (in Chinese).

[8] Zhang YZ, Fang HP, Zhu LS, et al. Construction of conceptual framework for sharing decision of cancer patients based on clinical decision theory. CHINESE NURSING RESEARCH 2020;34(01):136-41 (in Chinese).

[9] Li YZ. A Study on Status Quo and Influencing Factors of Thyroid Cancer Patients’Participation in Treatment Decision-Making. 2019 (in Chinese).

[10] Bian W, Wan JL, Su J, et al. Experience of Shared Decision Making in Patients with Age-Related Macular Degeneration: A Qualitative Research. Hospital Administration Journal of Chinese People's Liberation Army 2019;26(11):1039-41 (in Chinese).

[11] Zheng HY, Yang LN, You TT, et al. The barriers and facilitators of breast cancer patients' participation in Shared Decision-Making: a descriptive qualitative study. Chinese Nursing Management 2020;20(10):1492-6 (in Chinese).

[12] Fu JQ, He LJ. Investigation and analysis of requirements to participate in medical decision making in patients with breast cancer. Chinese Journal of Modern Nursing 2016;22(13):1881-4 (in Chinese).

[13] Cai C, Fang HP, Liu HJ, et al. Participation of patients with breast neoplasms in decision-making regarding treatment and nursing care: a qualitative study. Modern Clinical Nursing 2020;19(09):26-31 (in Chinese).

[14] Wan JL, Bian W, Liu MQ, et al. Qualitative study on factors influencing co-decision in patients with wet age-related macular degeneration. Journal of Clinical and Pathological Research 2020;40(02):417-22 (in Chinese).

[15] Zhang LJ, Zhang QW, Wang HQ, et al. A Survey Analysis of the Feasibility of a Bidirectional Way for Encouraging Shared Decision-making between Physicians and Patients. Medicine & Philosophy(B) 2013;34(02):94-7 (in Chinese).

[16] Feng XM, Zou YS. Diabetic Patients’Attitudes towards the Patient Decision Aids in Shared Decision-making. Medicine & Philosophy 2021;42(10):62-6 (in Chinese).

[17] Yuan N, Liu CE, Yu L, et al. Status Quo of Participation of Surgery Patients in Surgical Decision-making and Its Influence Factors. Journal of Nursing（China） 2017;24(04):47-50 (in Chinese).

[18] Zhang ZH, Xie FL, Chen AP, et al. A qualitative study of the psychological experience and supportive needs of patients with advanced lung cancer participating in and implementing treatment decisions. Modern Clinical Nursing 2020;19(07):30-6 (in Chinese).

[19] Wang J, Liu YX, Wang RX, et al. Study on Participation in Medication Decision-making in Elderly Patients with Multiple Chronic Diseases in Wuhan. Medicine and Society 2021;34(01):46-50 (in Chinese).

[20] Xie MK, Yang QF, Que J, et al. Atrial Fibrillation Patients’Involvement in Anticoagulation Therapy Decision-Conflict: A Qualitative Study. Nursing Journal of Chinese People's Liberation Army 2020;37(12):18-21 (in Chinese).

[21] Liu QM, Yu Q, Le MX. Effect of Expectation and Ability of Patients with Inflammatory Bowel Disease Participating in Clinical Decision Making on their Satisfaction with Clinical Participation. Shanghai Nursing 2021;21(01):19-22 (in Chinese).

[22] Su J, Bian W, Zhou FJ, et al. Qualitative study of treatment decision experience in patients with enucleation of eyeball. Journal of Clinical and Pathological Research 2020;40(01):124-9 (in Chinese).

[23] Yang HL. Related factors affecting the desires of patients with schizophrenia to be involved in medical decision. Medical Journal of Chinese People's Health 2014;26(23):3-7 (in Chinese).

[24] Bai DL, Hou XT, Liu XH, et al. Expectation for participation in medical decision making among advanced cancer patients: a cross-sectional study. Journal of Nursing Science 2017;32(05):35-8 (in Chinese).

[25] C H, V P, Y W, et al. I am the person who knows myself best: Perception on shared decision-making among hospitalized people diagnosed with schizophrenia in China. Int J Ment Health Nurs 2020;29(5):846-55.

[26] Huang C, Plummer V, Lam L, et al. Shared decision-making in serious mental illness: A comparative study. PATIENT EDUC COUNS 2020;103(8):1637-44.

[27] Tang H, Wang S, Dong S, et al. Surgery decision conflict and its related factors among newly diagnosed early breast cancer patients in China: A cross-sectional study. Nurs Open 2021;8(5):2578-86 (in Chinese).

[28] Luo H, Liu G, Lu J, et al. Association of shared decision making with inpatient satisfaction: a cross-sectional study. BMC Med Inform Decis Mak 2021;21(1):25 (in Chinese).

[29] Wu Q. Study on Process, Influencing Factors and Information Processing Feature of Atrial Fibrillation Patient Engagement in Treatment Decision Making. 2019.

[30] Wu SB, Lv AL. Application Value of Shared Decision-making on Doctor-patient Communication in ICU. Medicine & Philosophy 2019;40(06):25-7 (in Chinese).

[31] Y M, Wang AL, Qiao C P, et al. Design and implementation of PICC informed consent mobile medical decision aid program for gynecological tumor patients. Journal of Nursing Science 2021;36(08):5-9 (in Chinese).

[32] Yang H. Effect of health care shared decision-making mode on functional exercise in patients with breast cancer after operation. Chinese Journal of Practical Nursing 2019;11):836-41 (in Chinese).

[33] Wu LY. Influence of Shared Decision Intervention on Treatment Decision Conflict and Decision Satisfaction of Patients with Coronary Heart Disease. Journal of Qilu Nursing 2020;26(1):9-12 (in Chinese).

[34] W Y, Li L, Lang CY. The effect analysis of shared decision-making nursing model on neurology patients. Journal of Wenzhou Medical University 2019;49(04):301-5 (in Chinese).

[35] Zhang D M, Zhao YL, Feng ZJ. The application value of shared decision nursing in rapid rehabilitation of knee arthroplasty. Modern Journal of Integrated Traditional Chinese and Western Medicine 2019;28(10):1117-20 (in Chinese).

[36] Qian YF, Zhang XF, Jin AX, et al. The effect of shared decision-making model on chemotherapy integrity rate and decision participation satisfaction of breast cancer patients. Journal of Nursing and Rehabilitation 2020;19(04):54-6 (in Chinese).

[37] Xie CF. Application and Research of Share Decision Making in Emergency Patients With Hypertension. China Health Standard Management 2021;12(02):139-42 (in Chinese).

[38] Du LY. Effects of shared decision-making rapid rehabilitation nursing on anxiety level and joint function recovery of patients with knee arthroplasty. Journal of Practical Medical Techniques 2021;28(05):714-6 (in Chinese).

[39] Liu XF, Xie JH, Yi YZ, et al. Application of shared decision making in rapid rehabilitation of congenital tibial pseudarthrosis in children. Modern Nurse 2020;27(10):93-6 (in Chinese).

[40] Shi RZ, Hao YX, Fan XY, et al. Development of Decision Aid with Ottawa Decision Support Framework for Patients with Implantable Cardioverter Defibrillator. Journal of Nursing(China) 2019;26(05):35-40 (in Chinese).

[41] Liu N, Li Q, Ji MM, et al. Effect of Medical Staff－Family Members Combined Intervention Based on Shared Decision on Gastric Cancer Patients. Journal of Qilu Nursing 2021;27(08):1-3 (in Chinese).

[42] Mou CY, Qu Y, Cao N. Effect of patient-participation decision-making model based on nurse-patient harmony vision on patients' decision intention and disease management in neurology department. Journal of Nurses Training 2019;34(23):2169-73 (in Chinese).

[43] Li Y, Qiao J, Wang Y. Application of doctor-patient SDM-based individualized management mode in T2DM patients receiving insulin therapy. Chinese Journal of Clinical Research 2019;32(11):1501-6 (in Chinese).

[44] Deng JX, Dong ZH, Yin ZF. The value of decision assistance in controlling risk factors of atherosclerotic cardiovascular disease in diabetic patients. Shanxi Medical Journal 2019;48(01):56-8 (in Chinese).

[45] Zhou JJ. The influence of shared decision perception intervention on patients with coronary heart disease under the guidance of goal control theory. International Medicine and Health Guidance News 2020;26(15):2323-5 (in Chinese).

[46] Zhang WJ, Zhang DY, Li HH, et al. Application of doctor-nurse-patient sharing decision-making in perioperative ERAS model for patients with esophageal cancer. Chinese Journal of Surgical Oncology 2020;12(05):493-7 (in Chinese).

[47] Chen Y, Duan XC, Zhang YJ. The Influence of Shared Decision-making on Negative Emotion, Compliance Behavior and Treatment Effect in Patients with Acne. Chinese Journal of Aesthetic Medicine 2021;30(03):156-9 (in Chinese).

[48] Wang YJ. Analysis of doctor-patient Shared Decision-making on prognosis of diabetes mellitus complicated with cerebral infarction. Chinese Journal of Urban and Rural Enterprise Hygiene 2019;34(04):192-4 (in Chinese).

[49] Ou YH, Lu T, Li YL. Application of doctor-patient SDM intervention model in patients with bipolar disorder. Modern Diagnosis and Treatment 2021;32(01):137-8 (in Chinese).

[50] Li YL, Luo WL, Chen SY, et al. Application of doctor-patient shared decision making intervention in schizophrenic patients with persistent auditory hallucinations. Journal of Qilu Nursing 2020;26(09):53-5 (in Chinese).

[51] Chen HH, Chen SY, Chen XZ. Application of doctor-patient SDM in preventing postoperative thrombosis in elderly patients with lower limb fracture surgery. Journal of Qilu Nursing 2020;26(24):78-80 (in Chinese).

[52] Liang QS, Du Y, Pan J, et al. Influences of shared decision making on schizophrenic treatment compliance. Journal of Clinical Psychosomatic Diseases 2015;5):116-8 (in Chinese).

[53] Zhao XM, Wu GH. Influence of diagnosis and treatment mode of doctor-patient joint decision-making on medication compliance and clinical efficacy of community patients with psychiatric disorders*. China Medical Engineering 2017;25(12):16-8 (in Chinese).

[54] Ding WY. Application of doctor-patient shared decision-making in hemodialysis patients. Modern Practical Medicine 2020;32(09):1124-6 (in Chinese).

[55] Meng Y, Gai BJ, Kan LL, et al. Effects of treatment decision aid on decision-making difficulties and anxiety,depression in patients with lung cancer. Chinese Journal of Modern Nursing 2018;24(14):1692-5 (in Chinese).

[56] Guo YR. Construction and Application of Decision Aids Program for Functional Exercise in Patients with Kinesiophobia of Unilateral Total Knee Arthroplasty. 2020 (in Chinese).

[57] Li Y. Construction and Application of Treatment Decision Aids for Early-Stage Primary Liver Cancer Patients. 2017 (in Chinese).

[58] Shi RZ. Development and Application of a Decision Aid for Implantable cardioverter defibrillator Candidates. 2019 (in Chinese).

[59] Wang ST, Ye Zh X, Pan ZY, et al. Design of Decision-making Platform for Treating Patients with Primary Liver Cancer. Hospital Administration Journal of Chinese People's Liberation Army 2021;28(02):129-33 (in Chinese).

[60] Huang R, Song X, Wu J, et al. Assessing the feasibility and quality of shared decision making in China: evaluating a clinical encounter intervention for Chinese patients. Patient Prefer Adherence 2016;10(2341-50.
