# Supplementary material for: Shared decision-making in healthcare in mainland China: a scoping review
Source: Front Public Health. 2023 Sep 7;11:1162993. doi: 10.3389/fpubh.2023.1162993 (PMC10513465; doi:10.3389/fpubh.2023.1162993)
Supplement: Supplementary file 4 [file Table_4.DOCX]

Appendix 3 Theories/models/frameworks use to guide SDM (13 of 60 studies reported) [1-13]

| **Theories/models/frameworks** | **No. of studies (n (%))** | **The role in research** |
| --- | --- | --- |
| Ottawa Decision Support Framework  [1, 2, 4-7, 10, 12] | 8(61.54%) | Guiding the construction of decision aids  Guiding the assessment of decision-making needs  Guiding the design of the interview outline |
| Models by Shay L.A and colleagues [8] | 1 (7.69%) | Analyzing of the relationship between patient outcomes |
| Four Boxes of clinical decision by Jonsen Albert and colleagues [9] | 1 (7.69%) | Guiding in facilitating patient decision-making participation  Guiding in building decision-making participation support systems |
| Core domain set of outcomes of Shared Decision Making Process by Toupin-April and colleagues [11] | 1 (7.69%) | Guiding the design of the interview outline |
| Interprofessional Shared Decision Making Model (IP-SDM Model) [11] | 1 (7.69%) | Analyzing data according to micro, meso, and macro factors |
| Naturalistic decision-making theory [13] | 1 (7.69%) | Guiding the design of the study, including the design of the interview outline  Guiding the interpretation of results |
| Dual-process theories [13] | 1 (7.69%) | Interpreting patient decision-making behavior  Providing the theoretical basis for the decision-making process |
| Prospect Theory [13] | 1 (7.69%) | Guiding analysis of information processing features in decision making  Interpreting patient decision-making behavior |
| Information Seeking Model [13] | 1 (7.69%) | Understanding and analyzing patient decision-making information needs and information-seeking behaviors |
| Expanded Conceptual Model of Health information Seeking Behaviors and the Use of Information for Health Care Decisions [13] | 1 (7.69%) | Interpreting patient health information seeking behaviors and the use of information for health care decisions |
| Interactive Decision-Making Framework [13] | 1 (7.69%) | Providing a basis for the selection of influencing factors for patient participation in treatment decisions |
| Promoting Action on Research Implementation in Health Services framework (PARIHS) [5] | 1 (7.69%) | Guiding the implementation of decision aids |
| Target control theory [3] | 1 (7.69%) | Ensuring smooth implementation of intervention programs |

**References**

[1] Y M, Wang AL, Qiao CP, et al. Design and implementation of PICC informed consent mobile medical decision aid program for gynecological tumor patients. Journal of Nursing Science 2021;36(08):5-9 (in Chinese) .

[2] Shi RZ, Hao YX, Fan XY, et al. Development of Decision Aid with Ottawa Decision Support Framework for Patients with Implantable Cardioverter Defibrillator. Journal of Nursing(China) 2019;26(05):35-40 (in Chinese) .

[3] Zhou JJ. The influence of shared decision perception intervention on patients with coronary heart disease under the guidance of goal control theory. International Medicine and Health Guidance News 2020;26(15):2323-5 (in Chinese) .

[4] Guo YR. Construction and Application of Decision Aids Program for Functional Exercise in Patients with Kinesiophobia of Unilateral Total Knee Arthroplasty. 2020 (in Chinese) .

[5] Li Y. Construction and Application of Treatment Decision Aids for Early-Stage Primary Liver Cancer Patients. 2017 (in Chinese) .

[6] Shi RZ. Development and Application of a Decision Aid for Implantable cardioverter defibrillator Candidates. 2019 (in Chinese) .

[7] Wang ST, Ye ZhX, Pan ZY, et al. Design of Decision-making Platform for Treating Patients with Primary Liver Cancer. Hospital Administration Journal of Chinese People's Liberation Army 2021;28(02):129-33 (in Chinese) .

[8] Ming J, Wei Y, He LY, et al. Corelation Analysis of Physician-Patient Shared Decision-making and the Adoption of New Medical Technologies. Chinese Hospital Management 2018;38(03):19-22 (in Chinese) .

[9] Zhang YZ, Fang HP, Zhu LS, et al. Construction of conceptual framework for sharing decision of cancer patients based on clinical decision theory. CHINESE NURSING RESEARCH 2020;34(01):136-41 (in Chinese) .

[10] Li YZ. A Study on Status Quo and Influencing Factors of Thyroid Cancer Patients’Participation in Treatment Decision-Making. 2019 (in Chinese) .

[11] Zheng HY, Yang LN, You TT, et al. The barriers and facilitators of breast cancer patients' participation in Shared Decision-Making: a descriptive qualitative study. Chinese Nursing Management 2020;20(10):1492-6 (in Chinese) .

[12] Cai C, Fang HP, Liu HJ, et al. Participation of patients with breast neoplasms in decision-making regarding treatment and nursing care: a qualitative study. Modern Clinical Nursing 2020;19(09):26-31 (in Chinese) .

[13] Wu Q. Study on Process, Influencing Factors and Information Processing Feature of Atrial Fibrillation Patient Engagement in Treatment Decision Making. 2019 (in Chinese) .
