# Supplementary material for: Shared decision-making in healthcare in mainland China: a scoping review
Source: Front Public Health. 2023 Sep 7;11:1162993. doi: 10.3389/fpubh.2023.1162993 (PMC10513465; doi:10.3389/fpubh.2023.1162993)
Supplement: Supplementary file 5 [file Table_5.docx]

Appendix **4.** Summary of the findings of each of the themes based on the ODSF Decisional Needs Coding Manual- 1) Decisional conflict, 2) Inadequate Knowledge, 3) Unrealistic expectations, 4)Unclear values, 5) Inadequate support/resources, 6) Complex decision characteristics, 7) Personal and clinical needs

The operational definitions of Ottawa Decision Support Framework (ODSF) Decisional Needs Coding Manual (the verstion of July 13, 2020) See in [https://decisionaid.ohri.ca/docs/develop/ODSF_Decisional_Needs_Coding_Manual.pdf](https://decisionaid.ohri.ca/docs/develop/ODSF_Decisional_Needs_Coding_Manual.pdf（）)

| **Manifestations (and Contributing Factors) for Each Decisional Needs** | **References** | **ODSF code** | **N(%) of studies (N=56)** |
| --- | --- | --- | --- |
| **Decisional conflict** | [1-16] | **1** | **16 (29%)** |
| unsure about the best course of action | [2, 5, 7, 14, 16] | 1.1 | 5 (8.93%) |
| worried about what could go wrong | [8, 10-12, 14-16] | 1.2 | 7 (12.50%) |
| questions what is desirable /important to them (personal values) when attempting decision | [14, 15] | 1.4 | 2 (3.57%) |
| feels distressed or upset when attempting decision | [1, 3, 4, 6, 9, 11, 14] | 1.5 | 7 (12.50%) |
| wavers between choices or changing one’s mind | [13, 14] | 1.6 | 2 (3.57%) |
| constantly thinks about decision/can’t get off mind | [12, 14] | 1.7 | 2 (3.57%) |
| Feeling physically stressed: muscle tension, rapid heartbeat, difficulty sleeping when trying to make decisions | — | 1.8 | — |
| **Inadequate Knowledge** | [1-4, 6-10, 12, 13, 16-41] | 2 | **37 (66.07%)** |
| unaware that a decision needs to be made | [2, 9, 17, 29, 34-37, 39] | 2.1 | 9 (16.07%) |
| don’t know (enough) about the health problem, condition, or situation to make a decision | [1, 3, 4, 6-9, 12, 13, 16-36, 38, 40, 41] | 2.2 | **33 (**58.93**%)** |
| don’t know (enough about) options | [10, 37, 39] | 2.3 | 3 (5.36%) |
| don’t know (enough about) benefits, harms/risks, and/or scientific uncertainties | — | 2.4 | — |
| **Unrealistic Expectations** | [3, 34, 42] | 3 | **3 (5.36%)** |
| don’t know chances of benefits, harms/risks for each option | [3, 34] | 3.1 | 2 (3.57%) |
| - perceptions of one’s outcome probabilities are not aligned with current evidence for similar people | [34] | 3.2 | 1 (1.79%) |
| - difficult believing that the outcome probabilities apply to them | [34, 42] | 3.3 | 2 (3.57%) |
| **Unclear Values** | [11, 29, 42] | 4 | **3 (5.36%)** |
| unclear about option features that are important to them | [11, 29, 42] | 4.1 | 3 (5.36%) |
| **Inadequate Support and Resources** | [1-7, 9-23, 25-37, 39-41, 43-55] | **5** | 51（91.07%） |
| **Social Pressure** | [10, 29, 31, 36] | **5.2** | **4 (7.14%)** |
| **Difficult decisional roles** | [11, 12, 14-16, 31, 34, 36, 40, 41, 43] | **5.3** | **11 (19.64%)** |
| Unclear about role in decision making | [11, 12, 15, 31, 34, 40, 41] | **5.3.1** | **7** (12.50%) |
| Difficulty involving family member in decision-making. | [14-16, 41, 43] | **5.3.3** | **5** (8.93%) |
| Difficult shared family deliberation on options | [14] | **5.3.4** | **1** (1.79%) |
| Difficulty deliberating with practitioner because the patient/family | [12, 15, 31, 36] | **5.3.5** | **4** (7.14%) |
| **Inadequate experience** | [32, 35, 54, 55] | **5.4** | **4 (7.14%)** |
| lacks experience | [32, 35, 54, 55] | **5.4.1** | **4** **(7.14%)** |
| **Inadequate self-efficacy** | [2, 4, 6, 7, 11, 12, 15-20, 22, 26, 28, 30, 32, 34, 36, 37, 39, 41, 51] | **5.5** | **23 (41%)** |
| **lacks belief/confidence in ability to participate in decision-making** | [2, 4, 6, 7, 12, 15-20, 22, 26, 28, 30, 32, 34, 36, 37, 39, 41, 51] | **5.5.1** | **22 (39.29%)** |
| **lacks** **beliefconfidence in ability to implement chosen option** | [11, 37] | **5.5.2** | **2** (3.57%) |
| **Inadequate motivation** | [35, 36] | **5.6** | 2 (3.57%) |
| **lacks the ability or** **skill to make a decision** | [15, 41, 53] | **5.7** | 3 (5.36%) |
| **Inadequate information** | [9, 12-14, 21, 23, 28, 34, 35, 41, 48, 55] | **5.8** | 12 (21%) |
| Lacks access to (did not receive) information. | [9, 12-14, 21, 23, 28, 41, 48, 55] | **5.8.1** | **10 (17.86%)** |
| poor quality information | [12-14, 34, 35] | **5.8.2** | **5** (8.93%) |
| **Inadequate advice** | [7, 10, 15, 28-30, 39] | **5.9** | 7 (12.50%) |
| - lacks advice from important others involved in the decision | [7, 15, 30] | **5.9.1** | **3** (5.36%) |
| poor quality advice from important others involved in the decision | [7, 10, 28, 29, 39] | **5.9.2** | **5** (8.93%) |
| **Inadequate emotional** **support** | [1, 3-7, 12, 14-16, 22, 29-31, 36, 37, 45, 50, 51, 53] | **5.10** | **20 (35.71%)** |
| **Inadequate instrumental help** | [1-4, 7, 17-22, 25, 27, 34, 43-50, 52, 53] | **5.11** | **24 (42.86%)** |
| lacks instrumental help | [1-4, 7, 17-22, 25, 27, 34, 43-50, 52, 53] | **5.11.1** | **24** (42.86%) |
| **Inadequate health and social services** | [7, 11, 15, 27, 29, 31, 34, 39] | **5.12** | 8 (14.29%) |
| lacks health & social services, specify | [7, 11, 15, 27, 29, 31, 34, 39] | **5.12.1** | 8 (14.29%) |
| **Inadequate financial assistance** | [9, 14, 15, 32, 33, 36, 37, 45, 54] | **5.13** | 9 (16.07%) |
| lacks financial assistance, specify | [9, 14, 15, 32, 33, 36, 37, 45, 54] | **5.13.1** | 9 (16.07%) |
| **Complex Decision Characteristics** | [10, 11, 15, 16, 34, 35, 38, 55] | **6** | **8(14.29%)** |
| **Difficult Decision Type** | [35, 55,56] | **6.1** | 3 (5.36%) |
| Multiple options | [35,56] | **6.1.1** | **2** (3.57%) |
| Unknown outcomes, specify | [55] | **6.1.2** | **1** (1.79%) |
| **Difficult Decision Timing** | [10, 11, 15, 16, 34, 38] | **6.2** | **6 (10.71%)** |
| Timing is urgent | [10, 11, 15, 38] | **6.2.1** | **4** (7.14%) |
| Decision needs to be made soon | [10] | **6.2.2** | **1** (1.79%) |
| Timing is delayed | [16, 34] | **6.2.3** | **2** (3.57%) |
| **Unreceptive Decisional Stage** | — | **6.3** | **—** |
| **Personal and Clinical Needs** | [2, 3, 6, 7, 11, 15, 17, 20, 28, 29, 32-34, 41, 44, 45, 50, 51,56] | **7** | **19（34%）** |
| **Personal Needs** | [7, 11, 15, 28, 32-34, 41, 44] | **7.1** | **9 (16.07%)** |
| Special needs due to patient’s personal characteristics, specify need/characteristic | [32] | **7.1.1** | **1** (1.79%) |
| Need for tailored decision support | [7, 11, 15, 28, 33, 34, 41, 44] | **7.1.2** | **8** (14.29%) |
| **Clinical Needs** | [2, 3, 6, 17, 20, 29, 45, 50, 51,56] | **7.2** | **10 (17.86%)** |
| Special needs due to patient’s clinical characteristics, specify need/characteristic | [2, 3, 6, 17, 20, 29, 45, 50, 51,56] | **7.2.1** | **10 (17.86%)** |

**References**

[1] Y M, Wang AL, Qiao CP, et al. Design and implementation of PICC informed consent mobile medical decision aid program for gynecological tumor patients. Journal of Nursing Science 2021;36(08):5-9 (in Chinese).

[2] Wu LY. Influence of Shared Decision Intervention on Treatment Decision Conflict and Decision Satisfaction of Patients with Coronary Heart Disease. Journal of Qilu Nursing 2020;26(1):9-12 (in Chinese).

[3] Zhang DM, Zhao YL, Feng ZJ. The application value of shared decision nursing in rapid rehabilitation of knee arthroplasty. Modern Journal of Integrated Traditional Chinese and Western Medicine 2019;28(10):1117-20 (in Chinese).

[4] Du LY. Effects of shared decision-making rapid rehabilitation nursing on anxiety level and joint function recovery of patients with knee arthroplasty. Journal of Practical Medical Techniques 2021;28(05):714-6 (in Chinese).

[5] Meng Y, Gai BJ, Kan LL, et al. Effects of treatment decision aid on decision-making difficulties and anxiety,depression in patients with lung cancer. Chinese Journal of Modern Nursing 2018;24(14):1692-5 (in Chinese).

[6] Guo YR. Construction and Application of Decision Aids Program for Functional Exercise in Patients with Kinesiophobia of Unilateral Total Knee Arthroplasty. 2020 (in Chinese).

[7] Li Y. Construction and Application of Treatment Decision Aids for Early-Stage Primary Liver Cancer Patients. 2017 (in Chinese).

[8] Zhang QW, Wan XL, Liu Y, et al. A Survey Analysis of Patients’Perceptions of Difficulties in Shared Clinical Decision-Making. Chinese Journal of Practical Nursing 2010;10(01):10-3 (in Chinese).

[9] Yuan YJ, Wu Y, Yan MQ. Study on the patients’preferences for involvement in surgical decision-making and influencing factors. Journal of Nursing Science 2014;29(10):23-5 (in Chinese).

[10] Bian W, Wan JL, Su J, et al. Experience of Shared Decision Making in Patients with Age-Related Macular Degeneration: A Qualitative Research. Hospital Administration Journal of Chinese People's Liberation Army 2019;26(11):1039-41 (in Chinese).

[11] Zheng HY, Yang LN, You TT, et al. The barriers and facilitators of breast cancer patients' participation in Shared Decision-Making: a descriptive qualitative study. Chinese Nursing Management 2020;20(10):1492-6 (in Chinese).

[12] Cai C, Fang HP, Liu HJ, et al. Participation of patients with breast neoplasms in decision-making regarding treatment and nursing care: a qualitative study. Modern Clinical Nursing 2020;19(09):26-31 (in Chinese).

[13] Zhang LJ, Zhang QW, Wang HQ, et al. A Survey Analysis of the Feasibility of a Bidirectional Way for Encouraging Shared Decision-making between Physicians and Patients. Medicine & Philosophy(B) 2013;34(02):94-7 (in Chinese).

[14] Xie MK, Yang QF, Que J, et al. Atrial Fibrillation Patients’Involvement in Anticoagulation Therapy Decision-Conflict: A Qualitative Study. Nursing Journal of Chinese People's Liberation Army 2020;37(12):18-21 (in Chinese).

[15] C H, V P, Y W, et al. I am the person who knows myself best: Perception on shared decision-making among hospitalized people diagnosed with schizophrenia in China. Int J Ment Health Nurs 2020;29(5):846-55.

[16] Tang H, Wang S, Dong S, et al. Surgery decision conflict and its related factors among newly diagnosed early breast cancer patients in China: A cross-sectional study. Nurs Open 2021;8(5):2578-86 (in Chinese).

[17] Yang H. Effect of health care shared decision-making mode on functional exercise in patients with breast cancer after operation. Chinese Journal of Practical Nursing 2019;11):836-41 (in Chinese).

[18] W Y, Li L, Lang CY. The effect analysis of shared decision-making nursing model on neurology patients. Journal of Wenzhou Medical University 2019;49(04):301-5 (in Chinese).

[19] Qian YF, Zhang XF, Jin AX, et al. The effect of shared decision-making model on chemotherapy integrity rate and decision participation satisfaction of breast cancer patients. Journal of Nursing and Rehabilitation 2020;19(04):54-6 (in Chinese).

[20] Mou CY, Qu Y, Cao N. Effect of patient-participation decision-making model based on nurse-patient harmony vision on patients' decision intention and disease management in neurology department. Journal of Nurses Training 2019;34(23):2169-73 (in Chinese).

[21] Deng JX, Dong ZH, Yin ZF. The value of decision assistance in controlling risk factors of atherosclerotic cardiovascular disease in diabetic patients. Shanxi Medical Journal 2019;48(01):56-8 (in Chinese).

[22] Chen Y, Duan XC, Zhang YJ. The Influence of Shared Decision-making on Negative Emotion, Compliance Behavior and Treatment Effect in Patients with Acne. Chinese Journal of Aesthetic Medicine 2021;30(03):156-9 (in Chinese).

[23] Ou YH, Lu T, Li YL. Application of doctor-patient SDM intervention model in patients with bipolar disorder. Modern Diagnosis and Treatment 2021;32(01):137-8 (in Chinese).

[24] Li YL, Luo WL, Chen SY, et al. Application of doctor-patient shared decision making intervention in schizophrenic patients with persistent auditory hallucinations. Journal of Qilu Nursing 2020;26(09):53-5 (in Chinese).

[25] Chen HH, Chen SY, Chen XZ. Application of doctor-patient SDM in preventing postoperative thrombosis in elderly patients with lower limb fracture surgery. Journal of Qilu Nursing 2020;26(24):78-80 (in Chinese).

[26] Liang QS, Du Y, Pan J, et al. Influences of shared decision making on schizophrenic treatment compliance. Journal of Clinical Psychosomatic Diseases 2015;5):116-8 (in Chinese).

[27] Shi RZ. Development and Application of a Decision Aid for Implantable cardioverter defibrillator Candidates. 2019 (in Chinese).

[28] Zhang HW, Hou XT, Bai DL, et al. The patients satisfaction with participation in medical and nursing decision making among cancer patients: a cross- sectional study. BMC Med Inform Decis Mak 2017;33(23):1805-9 (in Chinese).

[29] Guo Y, Wen XL, Xin X, et al. Inform Consent or Conceal the Truth: A Qualitative Study on the Real Experience of Sharing Decision-making between Doctors and Patients in Rectal Cancer Patients Undergoing Enterostomy. Chinese Medical Ethics 2020;33(01):80-4 (in Chinese).

[30] Zheng HX. Multi-dimensional research on the influence of maternal delivery decision from the perspective of shared decision making. 2019 (in Chinese).

[31] Zhang YZ, Fang HP, Zhu LS, et al. Construction of conceptual framework for sharing decision of cancer patients based on clinical decision theory. CHINESE NURSING RESEARCH 2020;34(01):136-41 (in Chinese).

[32] Li YZ. A Study on Status Quo and Influencing Factors of Thyroid Cancer Patients’Participation in Treatment Decision-Making. 2019 (in Chinese).

[33] Fu JQ, He LJ. Investigation and analysis of requirements to participate in medical decision making in patients with breast cancer. Chinese Journal of Modern Nursing 2016;22(13):1881-4 (in Chinese).

[34] Wan JL, Bian W, Liu MQ, et al. Qualitative study on factors influencing co-decision in patients with wet age-related macular degeneration. Journal of Clinical and Pathological Research 2020;40(02):417-22 (in Chinese).

[35] Feng XM, Zou YS. Diabetic Patients’Attitudes towards the Patient Decision Aids in Shared Decision-making. Medicine & Philosophy 2021;42(10):62-6 (in Chinese).

[36] Yuan N, Liu CE, Yu L, et al. Status Quo of Participation of Surgery Patients in Surgical Decision-making and Its Influence Factors. Journal of Nursing（China） 2017;24(04):47-50 (in Chinese).

[37] Zhang ZH, Xie FL, Chen AP, et al. A qualitative study of the psychological experience and supportive needs of patients with advanced lung cancer participating in and implementing treatment decisions. Modern Clinical Nursing 2020;19(07):30-6 (in Chinese).

[38] Wang J, Liu YX, Wang RX, et al. Study on Participation in Medication Decision-making in Elderly Patients with Multiple Chronic Diseases in Wuhan. Medicine and Society 2021;34(01):46-50 (in Chinese).

[39] Liu QM, Yu Q, Le MX. Effect of Expectation and Ability of Patients with Inflammatory Bowel Disease Participating in Clinical Decision Making on their Satisfaction with Clinical Participation. Shanghai Nursing 2021;21(01):19-22 (in Chinese).

[40] Bai DL, Hou XT, Liu XH, et al. Expectation for participation in medical decision making among advanced cancer patients: a cross-sectional study. Journal of Nursing Science 2017;32(05):35-8 (in Chinese).

[41] Huang C, Plummer V, Lam L, et al. Shared decision-making in serious mental illness: A comparative study. PATIENT EDUC COUNS 2020;103(8):1637-44.

[42] Zhao Y, Zhang Q, Liang LZ. Study on the Theory and Practical Problem of“Patient Participation”in Clinical Decision Making. Chinese Medical Ethics 2018;31(06):799-803 (in Chinese).

[43] Wu SB, Lv AL. Application Value of Shared Decision-making on Doctor-patient Communication in ICU. Medicine & Philosophy 2019;40(06):25-7 (in Chinese).

[44] Xie CF. Application and Research of Share Decision Making in Emergency Patients With Hypertension. China Health Standard Management 2021;12(02):139-42 (in Chinese).

[45] Liu XF, Xie JH, Yi YZ, et al. Application of shared decision making in rapid rehabilitation of congenital tibial pseudarthrosis in children. Modern Nurse 2020;27(10):93-6 (in Chinese).

[46] Shi RZ, Hao YX, Fan XY, et al. Development of Decision Aid with Ottawa Decision Support Framework for Patients with Implantable Cardioverter Defibrillator. Journal of Nursing(China) 2019;26(05):35-40 (in Chinese).

[47] Liu N, Li Q, Ji MM, et al. Effect of Medical Staff－Family Members Combined Intervention Based on Shared Decision on Gastric Cancer Patients. Journal of Qilu Nursing 2021;27(08):1-3 (in Chinese).

[48] Li Y, Qiao J, Wang Y. Application of doctor-patient SDM-based individualized management mode in T2DM patients receiving insulin therapy. Chinese Journal of Clinical Research 2019;32(11):1501-6 (in Chinese).

[49] Zhou JJ. The influence of shared decision perception intervention on patients with coronary heart disease under the guidance of goal control theory. International Medicine and Health Guidance News 2020;26(15):2323-5 (in Chinese).

[50] Zhang WJ, Zhang DY, Li HH, et al. Application of doctor-nurse-patient sharing decision-making in perioperative ERAS model for patients with esophageal cancer. Chinese Journal of Surgical Oncology 2020;12(05):493-7 (in Chinese).

[51] Ding WY. Application of doctor-patient shared decision-making in hemodialysis patients. Modern Practical Medicine 2020;32(09):1124-6 (in Chinese).

[52] Huang R, Song X, Wu J, et al. Assessing the feasibility and quality of shared decision making in China: evaluating a clinical encounter intervention for Chinese patients. Patient Prefer Adherence 2016;10(2341-50.

[53] Su J, Bian W, Zhou FJ, et al. Qualitative study of treatment decision experience in patients with enucleation of eyeball. Journal of Clinical and Pathological Research 2020;40(01):124-9 (in Chinese).

[54] Yang HL. Related factors affecting the desires of patients with schizophrenia to be involved in medical decision. Medical Journal of Chinese People's Health 2014;26(23):3-7 (in Chinese).

[55] Luo H, Liu G, Lu J, et al. Association of shared decision making with inpatient satisfaction: a cross-sectional study. BMC Med Inform Decis Mak 2021;21(1):25 (in Chinese).

[56] WuQing. Study on Process, Influencing Factors and Information Processing Feature of Atrial Fibrillation Patient Engagement in Treatment Decision Making. 2019 (in Chinese).
