# Supplementary material for: Shared decision-making in healthcare in mainland China: a scoping review
Source: Front Public Health. 2023 Sep 7;11:1162993. doi: 10.3389/fpubh.2023.1162993 (PMC10513465; doi:10.3389/fpubh.2023.1162993)
Supplement: Supplementary file 6 [file Table_6.DOCX]

Appendix 5.

**The SDM implementation process**

Twenty-five research mentioned "equipoise" (25/31,80.65%) and we classified this theme into communicating the condition (9/31,29.03%) ^[78, 80-82, 84, 85, 95, 102, 103]^ , evaluating the patient (13/31,41.94%) ^[46, 53, 55, 56, 73-75, 77, 79, 80, 87, 90, 91]^, providing options (8/31,25.81%) ^[46, 78, 81, 84, 85, 87, 95, 96, 102]^, developing plans (selection of tools) (4/31,12.90%) ^[72, 76, 88, 91]^, clarifying decision-making goals (1/31,3.23%) [53]. 24 studies (24/31, 77.42%) ^[46, 52, 53, 55, 56, 71, 73-75, 78-80, 82, 84-87, 89-91, 95, 96, 102, 103]^, mentioned "exchange of information" we divided this theme into health education (one-way) (7/31, 22.58%) ^[73, 74, 85-87, 91, 96]^, sharing information (14/31, 45.16%) ^[46, 52, 53, 71, 75, 78-80, 82, 84, 90, 95, 102, 103]^, feedback (2/31, 6.45%) ^[52, 86]^, discussion with interventions (5/31,16.13%) ^[53, 55, 89, 90, 96]^.

Sixteen studies(16/31,51.61%) ^[46, 52, 53, 71, 78, 79, 82, 84-86, 89, 90, 95, 96, 102, 103]^ mentioned "values clarification" , and we divided this topic into acquiring patients’ perspectives (15/ 31,48.39%) ^[46, 52, 53, 71, 78, 79, 82, 84-86, 89, 90, 95, 96, 102, 103]^, introducing of risk-benefit (9/31,29.03%) ^[43, 71, 82, 86, 90, 95, 96, 102, 100]^, decision making (4/31,12.90%) ^[52, 78, 79, 84]^.

Eight studies referred to "feasibility of the options" (8/31,25.81%) ^[46, 71, 78, 82, 84, 95, 102]^, which can be divided into reaching consensus (5/31,16.13%) ^[46, 82, 84, 86, 95]^, making recommendations (alternatives) (2/31,6.45%) ^[71, 78]^, restating options (4/31,12.90%) ^[46, 76, 83, 102]^, reassessing the patient's situation (3/31,9.68%) ^[73, 82, 102]^ .

Seven studies(7/31,22.58%) ^[53, 77, 78, 84, 85, 96, 102]^mentioned the "Preferred Choice" of patients, which can be divided into consulting choice (4/31,12.90%) ^[53, 78, 84, 102]^, reaching consensus (5/31,16.13%) ^[74, 75, 81, 82, 93]^.

Ten studies (10/31,32.26%) ^[46, 53, 73-75, 75, 84, 85, 93, 102]^ mentioned patients' "Actual Choice" , classified as clarifying decision-making (7/31,22.58%) ^[46, 53, 73-75, 96, 102]^ , signing treatment Consent (1/31,3.23%) ^[85]^, consulting decision-making (3/31,9.68%) [46, 78, 84].

Seven studies (7/31,22.58%) ^[46, 71, 74, 77, 88-90]^ mentioned "Implementation" , which can be divided into follow-up (3/31,9.68%) ^[73, 77, 89]^, implementation of decision-making (4/31,12.90%) ^[^^46, 71, 88, 90]^.

Five studies (5/31,16.13%) ^[55, 72, 73, 76, 89]^ mentioned "health outcomes" which can be classified as decision-related evaluations (4/31,12.90%) ^[46, 71, 88, 90]^ and efficacy evaluations (1/31,3.23%) ^[70]^.

**The process of developing the SDM support tools**

Corresponding to the standard, only 8 of these steps could be reflected in our study. "Define the decision" was mentioned in only one study (1/30, 3.33%)^[27]^. "Bring together a multidisciplinary group and include the person" were reflected in four studies (4/30, 13.33%)^[29, 53, 68, 73]^; three studies (3/31,10.00%)^[27, 43, 73]^ mentioned"define the equality and diversity considerations"", including clarifying needs by assessing patients ^[27, 43]^, and assessing experts ^[73]^; two studies (2/31, 6.67%) ^[27, 29]^ mentioned "define the criteria of the decision aid". “Analyse evidence-based sources” were mentioned in five studies (5/31,16.67%)^[29, 52, 53, 82, 86]^ , of which three^[53, 82, 86]^ mentioned guidelines and two^[29, 52]^ did not mention specific sources ; Four studies mentioned “determine the most appropriate tool for the decision” (4/31,13.33%)^[53, 68, 82, 86]^ , while one ^[68]^ mentioned the presentation format (video, website), three ^[53, 82, 86]^did not mention the specific format; five mentioned content writing (5/30,16.67%), two^[27, 73]^included experts meetings, and four ^[27, 43, 53, 82]^ studies included expert inquiries; five studies mentioned stakeholder evaluation (5/30,16.67%) including expert evaluation ^[29, 53, 73]^, and patient evaluation ^[29, 43, 53, 86]^, of which only one study (1/30,3.33%) ^[29]^mentioned IPDAS international standards.

**This appendix provides reference numbers that correspond to those in the text. Please refer to the number above for detailed information on each reference.**
