# Supplementary material for: Shared decision-making in healthcare in mainland China: a scoping review
Source: Front Public Health. 2023 Sep 7;11:1162993. doi: 10.3389/fpubh.2023.1162993 (PMC10513465; doi:10.3389/fpubh.2023.1162993)
Supplement: Supplementary file 7 [file Table_7.DOCX]

Appendix 6.

**The outcomes of participation in SDM in China**

| **Primary theme** | **Quantitative outcome indicators** | **No. of studies (n (%))** |
| --- | --- | --- |
| Affective-cognitive outcomes(36/60,60.00%)[1-36] | **Understanding** [1-10, 15-32, 35]  Cognitive level scale (self-made)[1-5, 15-18]  Control Preference Scale (CPS) [1, 6, 7, 17, 19-24, 27, 28, 32]  Decision Conflict Scale (DCS) [8-10, 25]  Decision Regret Scale (DRS) [10]  Autonomy preference index (API) [1, 19]  Patient Perceived Engagement Scale (PPES)[26]  Patient's willingness to participate in clinical decision-making (Self-made) [15, 16, 28-31]  Patient Participation Attitude Scale (PPAS) [32]  Clinical Decision-Making Style Scale (CDMS)[35]  All Aspects of Health Literacy Scale ( AAHLS) [32] | **(29/60,48.33%)** |
|  | **Satisfaction**[1, 4, 7, 11-14, 17, 19, 22, 23, 32-35]  Decision-making participation satisfaction scale [17, 19, 23, 32-35]  Patient Satisfaction Scale [1, 4, 7, 11-14]  Patient Participation in Clinical Decision-Making Scale [22] | **(15/60,25.00%)** |
|  | **Trust** [18, 32]  Physician-Patient Relationship Scale [18]  Patient Participation Attitude Scale [10] | (**2/60,3.33% )** |
| Behavioral outcomes (**22/60,36.67.%)**  [2, 4, 5, 7, 8, 10, 18, 20, 22, 23, 25, 27, 35, 37-45] | **Adherence**[2, 4, 7, 8, 10, 37-42]  The 0bserving Patient Invo—lve—ment Scale (OPTION scale) [8, 38]  Aitken procrastination inventory (API) [7]  Decision-making efficiency for patients and nurses [7]  Shared Decision-Making Questionnaire (SDM-Q-9) [39]  Readiness to Make Decisions Scale (C-PrepDM) [10]  Functional Exercise Adherence Scale after Breast Cancer Surgery[37]  8-item Morisky medication adherence scale (MMAS-8) [39]  Medical compliance behavior questionnaire (self-administered) [2, 4, 40-42]  Exercise Adherence Rating Scale (EARS)[38] | **(11/60,18.33%)** |
|  | **Treatment decision** [10]  Facilitation of patient involvement scale (FPI) [10]  Decision Readiness Scale (C-PrepDM) [10] | (1/60,1.67%) |
|  | **Health behaviors**[5, 18, 20, 22, 23, 25, 27, 35, 39, 43-45]  Control Preference Scale (CPS)[18, 27]  Patient Involvement in Clinical Decision-Making Power Scale [20, 22, 23]  Patient Participation Competence Scale (PPCS)[25]  Patient Involvement in Decision-Making Scale [45]  Family Engagement Self-Reporting Scale [35]  SDSCA-6 Scale[43]  Scale of Health Behavior (SRAHP) [39]  Self-Care Competency Scale (ESCA)[5, 44]  Kamofsky Performance Status (KPS) [27] | (12/60,20.00% ) |
| Health outcomes(**25/60,41.67%)**[1-6, 9, 11, 13, 14, 18, 26, 32, 37-42, 44, 46-50] | **Symptom reduction**[1-6, 9-11, 13, 14, 26, 37, 38, 40-42, 44, 46-49, 51]  CHA2DS2VASC Score and EHRA Symptom Classification [32]  Dability of Arm-Shoulder-Hand (DASH)[37]  Oral complaints grading scale [13, 46]  American Hospital for Special Surgery Knee Rating Scale (HSS) [38, 47]  Stroke impairment assessment set (SIAS)[1]  Acne lesion score [2]  National Institutes of Health Stroke Scale (NIHSS) [48]  Wechsler Memory Scale (WMS) [44]  Wechsler Intelligence Scale (WISC-Ⅳ)[44]  Treatment Emergent Symptom Scale (TESS) [3]  Incidence of DVT [4]  Global Assessment Scale,GAS [41]  Brief Psychiatric Rating Scale (BPRS) [42]  Social Function Deficit Screening Scale (SDSS)[42]  Functional Assessment of Chronic Illness Treatment - Fatigue Scale (FACIT-F) [5]  Tampa Scale of Kinesiophobia (TSK) [38]  Maternal anxiety levels before delivery [26]  Self-rating Anxiety Scale (SAS) [2, 11, 40, 47, 49]  Child Anxiety Scale [13]  Self-rating Depression Scale (SDS) [2, 40]  Hamilton Depression Inventory (HAMD) [4, 14]  Hospital Anxiety and Depression Scale (HADS)[6, 9, 10, 38] | **(23/60, 38.33%)** |
|  | **Quality of life**[5, 11, 14, 18, 26, 37, 39]  Patient quality of life scores (SF-36)[11, 14]  Functional Assessmentof CancerTherapy-Breast Quality of Life Instrument (FACT-B)[37]  Minnesota Cardiac Insufficiency Quality of Life Scale (MLHFQ)[39]  Kidney Disease Quality of Life short Form 1.3 (KDQOL-SFTM 1.3) [5]  Family Social Support Status Scale [18, 26] | ( **7/60,11.67%)** |
|  | **Physiological measures** [11, 48, 50]  Physiological and biochemical indicators[43, 48, 50]  90-day mortality and place of death [11] | (**3/60,5.00%)** |
| Length of stay and cost of hospitalization[11, 13] | | (2/60,3.33%) |

**References**

[1] Mou CY, Qu Y, Cao N. Effect of patient-participation decision-making model based on nurse-patient harmony vision on patients' decision intention and disease management in neurology department. Journal of Nurses Training 2019;34(23):2169-73 (in Chinese).

[2] Chen Y, Duan XC, Zhang YJ. The Influence of Shared Decision-making on Negative Emotion, Compliance Behavior and Treatment Effect in Patients with Acne. Chinese Journal of Aesthetic Medicine 2021;30(03):156-9 (in Chinese).

[3] Li YL, Luo WL, Chen SY, et al. Application of doctor-patient shared decision making intervention in schizophrenic patients with persistent auditory hallucinations. Journal of Qilu Nursing 2020;26(09):53-5 (in Chinese).

[4] Chen HH, Chen SY, Chen XZ. Application of doctor-patient SDM in preventing postoperative thrombosis in elderly patients with lower limb fracture surgery. Journal of Qilu Nursing 2020;26(24):78-80 (in Chinese).

[5] Ding WY. Application of doctor-patient shared decision-making in hemodialysis patients. Modern Practical Medicine 2020;32(09):1124-6 (in Chinese).

[6] Y M, Wang AL, Qiao CP, et al. Design and implementation of PICC informed consent mobile medical decision aid program for gynecological tumor patients. Journal of Nursing Science 2021;36(08):5-9 (in Chinese).

[7] W Y, Li L, Lang CY. The effect analysis of shared decision-making nursing model on neurology patients. Journal of Wenzhou Medical University 2019;49(04):301-5 (in Chinese).

[8] Wu LY. Influence of Shared Decision Intervention on Treatment Decision Conflict and Decision Satisfaction of Patients with Coronary Heart Disease. Journal of Qilu Nursing 2020;26(1):9-12 (in Chinese).

[9] Meng Y, Gai BJ, Kan LL, et al. Effects of treatment decision aid on decision-making difficulties and anxiety,depression in patients with lung cancer. Chinese Journal of Modern Nursing 2018;24(14):1692-5 (in Chinese).

[10] Li Y. Construction and Application of Treatment Decision Aids for Early-Stage Primary Liver Cancer Patients. 2017 (in Chinese).

[11] Wu SB, Lv AL. Application Value of Shared Decision-making on Doctor-patient Communication in ICU. Medicine & Philosophy 2019;40(06):25-7 (in Chinese).

[12] Xie CF. Application and Research of Share Decision Making in Emergency Patients With Hypertension. China Health Standard Management 2021;12(02):139-42 (in Chinese).

[13] Liu XF, Xie JH, Yi YZ, et al. Application of shared decision making in rapid rehabilitation of congenital tibial pseudarthrosis in children. Modern Nurse 2020;27(10):93-6 (in Chinese).

[14] Zhang WJ, Zhang DY, Li HH, et al. Application of doctor-nurse-patient sharing decision-making in perioperative ERAS model for patients with esophageal cancer. Chinese Journal of Surgical Oncology 2020;12(05):493-7 (in Chinese).

[15] Zhang LJ, Zhang QW, Wang HQ, et al. A Survey Analysis of the Feasibility of a Bidirectional Way for Encouraging Shared Decision-making between Physicians and Patients. Medicine & Philosophy(B) 2013;34(02):94-7 (in Chinese).

[16] Zhao Y, Zhang Q, Liang LZ. Study on the Theory and Practical Problem of“Patient Participation”in Clinical Decision Making. Chinese Medical Ethics 2018;31(06):799-803 (in Chinese).

[17] Zhang QW, Wan XL, Liu Y, et al. A Survey Analysis of Patients’Perceptions of Difficulties in Shared Clinical Decision-Making. Chinese Journal of Practical Nursing 2010;10(01):10-3 (in Chinese).

[18] Zhang YZ, Fang HP, Zhu LS, et al. Construction of conceptual framework for sharing decision of cancer patients based on clinical decision theory. CHINESE NURSING RESEARCH 2020;34(01):136-41 (in Chinese).

[19] Yuan YJ, Wu Y, Yan MQ. Study on the patients’preferences for involvement in surgical decision-making and influencing factors. Journal of Nursing Science 2014;29(10):23-5 (in Chinese).

[20] Yuan N, Liu CE, Yu L, et al. Status Quo of Participation of Surgery Patients in Surgical Decision-making and Its Influence Factors. Journal of Nursing（China） 2017;24(04):47-50 (in Chinese).

[21] Fu JQ, He LJ. Investigation and analysis of requirements to participate in medical decision making in patients with breast cancer. Chinese Journal of Modern Nursing 2016;22(13):1881-4 (in Chinese).

[22] Liu QM, Yu Q, Le MX. Effect of Expectation and Ability of Patients with Inflammatory Bowel Disease Participating in Clinical Decision Making on their Satisfaction with Clinical Participation. Shanghai Nursing 2021;21(01):19-22 (in Chinese).

[23] Zhang HW, Hou XT, Bai DL, et al. The patients satisfaction with participation in medical and nursing decision making among cancer patients: a cross- sectional study. BMC Med Inform Decis Mak 2017;33(23):1805-9 (in Chinese).

[24] Bai D L, Hou XT, Liu XH, et al. Expectation for participation in medical decision making among advanced cancer patients: a cross-sectional study. Journal of Nursing Science 2017;32(05):35-8 (in Chinese).

[25] Tang H, Wang S, Dong S, et al. Surgery decision conflict and its related factors among newly diagnosed early breast cancer patients in China: A cross-sectional study. Nurs Open 2021;8(5):2578-86 (in Chinese).

[26] Zheng HX. Multi-dimensional research on the influence of maternal delivery decision from the perspective of shared decision making. 2019 (in Chinese).

[27] Li YZ. A Study on Status Quo and Influencing Factors of Thyroid Cancer Patients’ Participation in Treatment Decision-Making. 2019 (in Chinese).

[28] Ming J, Wei Y, He LY, et al. Corelation Analysis of Physician-Patient Shared Decision-making and the Adoption of New Medical Technologies. Chinese Hospital Management 2018;38(03):19-22 (in Chinese).

[29] Feng XM, Zou YS. Diabetic Patients’Attitudes towards the Patient Decision Aids in Shared Decision-making. Medicine & Philosophy 2021;42(10):62-6 (in Chinese).

[30] Yang HL. Related factors affecting the desires of patients with schizophrenia to be involved in medical decision. Medical Journal of Chinese People's Health 2014;26(23):3-7 (in Chinese).

[31] Luo H, Liu G, Lu J, et al. Association of shared decision making with inpatient satisfaction: a cross-sectional study. BMC Med Inform Decis Mak 2021;21(1):25 (in Chinese).

[32] Wu Q. Study on Process, Influencing Factors and Information Processing Feature of Atrial Fibrillation Patient Engagement in Treatment Decision Making. 2019 (in Chinese).

[33] Guo Y, Wen XL, Xin X, et al. Inform Consent or Conceal the Truth: A Qualitative Study on the Real Experience of Sharing Decision-making between Doctors and Patients in Rectal Cancer Patients Undergoing Enterostomy. Chinese Medical Ethics 2020;33(01):80-4 (in Chinese).

[34] Wan JL, Bian W, Liu MQ, et al. Qualitative study on factors influencing co-decision in patients with wet age-related macular degeneration. Journal of Clinical and Pathological Research 2020;40(02):417-22 (in Chinese).

[35] Huang C, Plummer V, Lam L, et al. Shared decision-making in serious mental illness: A comparative study. PATIENT EDUC COUNS 2020;103(8):1637-44.

[36] Zhang ZH, Xie FL, Chen AP, et al. A qualitative study of the psychological experience and supportive needs of patients with advanced lung cancer participating in and implementing treatment decisions. Modern Clinical Nursing 2020;19(07):30-6 (in Chinese).

[37] Yang H. Effect of health care shared decision-making mode on functional exercise in patients with breast cancer after operation. Chinese Journal of Practical Nursing 2019;11):836-41 (in Chinese).

[38] Guo YR. Construction and Application of Decision Aids Program for Functional Exercise in Patients with Kinesiophobia of Unilateral Total Knee Arthroplasty. 2020 (in Chinese).

[39] Zhou JJ. The influence of shared decision perception intervention on patients with coronary heart disease under the guidance of goal control theory. International Medicine and Health Guidance News 2020;26(15):2323-5 (in Chinese).

[40] Liu N, Li Q, Ji MM, et al. Effect of Medical Staff－Family Members Combined Intervention Based on Shared Decision on Gastric Cancer Patients. Journal of Qilu Nursing 2021;27(08):1-3 (in Chinese).

[41] Liang QS, Du Y, Pan J, et al. Influences of shared decision making on schizophrenic treatment compliance. Journal of Clinical Psychosomatic Diseases 2015;5):116-8 (in Chinese).

[42] Zhao XM, Wu GH. Influence of diagnosis and treatment mode of doctor-patient joint decision-making on medication compliance and clinical efficacy of community patients with psychiatric disorders*. China Medical Engineering 2017;25(12):16-8 (in Chinese).

[43] Li Y, Qiao J, Wang Y. Application of doctor-patient SDM-based individualized management mode in T2DM patients receiving insulin therapy. Chinese Journal of Clinical Research 2019;32(11):1501-6 (in Chinese).

[44] Ou YH, Lu T, Li YL. Application of doctor-patient SDM intervention model in patients with bipolar disorder. Modern Diagnosis and Treatment 2021;32(01):137-8 (in Chinese).

[45] Wang J, Liu YX, Wang RX, et al. Study on Participation in Medication Decision-making in Elderly Patients with Multiple Chronic Diseases in Wuhan. Medicine and Society 2021;34(01):46-50 (in Chinese).

[46] Qian YF, Zhang XF, Jin AX, et al. The effect of shared decision-making model on chemotherapy integrity rate and decision participation satisfaction of breast cancer patients. Journal of Nursing and Rehabilitation 2020;19(04):54-6 (in Chinese).

[47] Du LY. Effects of shared decision-making rapid rehabilitation nursing on anxiety level and joint function recovery of patients with knee arthroplasty. Journal of Practical Medical Techniques 2021;28(05):714-6 (in Chinese).

[48] Wang YJ. Analysis of doctor-patient Shared Decision-making on prognosis of diabetes mellitus complicated with cerebral infarction. Chinese Journal of Urban and Rural Enterprise Hygiene 2019;34(04):192-4 (in Chinese).

[49] Zhang DM, Zhao YL, Feng ZJ. The application value of shared decision nursing in rapid rehabilitation of knee arthroplasty. Modern Journal of Integrated Traditional Chinese and Western Medicine 2019;28(10):1117-20 (in Chinese).

[50] Deng JX, Dong ZH, Yin ZF. The value of decision assistance in controlling risk factors of atherosclerotic cardiovascular disease in diabetic patients. Shanxi Medical Journal 2019;48(01):56-8 (in Chinese).

[51] F L, Tang CL, Ke X, et al. Study on the reliability and validity evaluation of the questionnaire of patients' participation in decision-making in the application of new medical technology. Chinese hospital 2018;22(10):4-7 (in Chinese).
